# The CIN4 Chromosomal Instability qPCR Classifier Defines Tumor Aneuploidy and Stratifies Outcome in Grade 2 Breast Cancer

**DOI:** 10.1371/journal.pone.0056707

**Published:** 2013-02-26

**Authors:** Attila Marcell Szász, Qiyuan Li, Aron C. Eklund, Zsófia Sztupinszki, Andrew Rowan, Anna-Mária Tőkés, Borbála Székely, András Kiss, Miklós Szendrői, Balázs Győrffy, Zoltán Szállási, Charles Swanton, Janina Kulka

**Affiliations:** 1 2nd Department of Pathology, Semmelweis University, Budapest, Hungary; 2 Center for Biological Sequence Analysis, Department of Systems Biology, Technical University of Denmark, Lyngby, Denmark; 3 Hungarian Academy of Sciences, Research Laboratory for Pediatrics and Nephrology, 1st Department of Pediatrics, Semmelweis University, Budapest, Hungary; 4 Cancer Research UK, London Research Institute, Translational Cancer Therapeutics Laboratory, London, United Kingdom; 5 Hungarian Academy of Sciences, MTA-SE Tumour Progression Research Group, 2nd Department of Pathology, Semmelweis University, Budapest, Hungary; 6 Department of Orthopaedics, Semmelweis University, Budapest, Hungary; 7 Informatics Program, Children's Hospital Boston, Harvard Medical School, Boston, Massachusetts, United States of America; 8 University College London Cancer Institute, London, United Kingdom; 9 Department of Pathology, Buda MÁV Hospital, Budapest, Hungary; Harvard School of Public Health, United States of America

## Abstract

**Purpose:**

Quantifying chromosomal instability (CIN) has both prognostic and predictive clinical utility in breast cancer. In order to establish a robust and clinically applicable gene expression-based measure of CIN, we assessed the ability of four qPCR quantified genes selected from the 70-gene Chromosomal Instability (CIN70) expression signature to stratify outcome in patients with grade 2 breast cancer.

**Methods:**

AURKA, FOXM1, TOP2A and TPX2 (CIN4), were selected from the CIN70 signature due to their high level of correlation with histological grade and mean CIN70 signature expression *in silico*. We assessed the ability of CIN4 to stratify outcome in an independent cohort of patients diagnosed between 1999 and 2002. 185 formalin-fixed, paraffin-embedded (FFPE) samples were included in the qPCR measurement of CIN4 expression. In parallel, ploidy status of tumors was assessed by flow cytometry. We investigated whether the categorical CIN4 score derived from the CIN4 signature was correlated with recurrence-free survival (RFS) and ploidy status in this cohort.

**Results:**

We observed a significant association of tumor proliferation, defined by Ki67 and mitotic index (MI), with both CIN4 expression and aneuploidy. The CIN4 score stratified grade 2 carcinomas into good and poor prognostic cohorts (mean RFS: 83.8±4.9 and 69.4±8.2 months, respectively, p = 0.016) and its predictive power was confirmed by multivariate analysis outperforming MI and Ki67 expression.

**Conclusions:**

The first clinically applicable qPCR derived measure of tumor aneuploidy from FFPE tissue, stratifies grade 2 tumors into good and poor prognosis groups.

## Introduction

Chromosomal instability (CIN) is a key determinant of biological behavior of breast cancer [1], yet remains challenging to determine using high throughput methodologies [2]. We and others have shown that CIN may play a role in determining response to taxane treatment in ovarian cancer [3] and intrinsic multidrug resistance in colon cancer [4] and it also appears to be an important determinant of breast cancer prognosis [5], . While direct determination of CIN by counting centromeres in a sufficient number of breast cancer cells or measuring DNA index is possible [7], it is technically challenging and time-consuming [8], [9]. Consequently, a simple measure of CIN, based on for example qPCR measurement of a handful of genes, would greatly facilitate its introduction into general oncological practice. Therefore, we decided to investigate whether a clinically implementable a qPCR-based gene expression based CIN measure could be created. The ability of such signatures to reflect CGH based genomic instability has been previously demonstrated [5].

The potential utility of a gene expression based measure of CIN is further emphasized by its complex relationship with histological grade [10]. It has been known for many years that grade 2 tumors classified by the Nottingham grading system display heterogeneous characteristics in terms of clinical outcome [11], [12]. It has also been shown that relatively simple gene expression based methods were able to stratify grade 2 cases into low risk and high risk patients in a robust fashion [13]. Genes intimately involved with CIN prominently featured in such gene expression signatures, but it has become clear recently that quantifying ploidy has improved prognostic potential than stratifying intermediate histological grade in terms of clinical outcome. The vast majority of hormone receptor negative cases (ER-/PgR-/HER2-) fall into histological grade 3 category [14], but even within those, the level of CIN may define subgroups of patients with distinct clinical outcomes [5]. Therefore in this study, we determined a minimal gene set that seems to capture the information contained in the previously published CIN70 signature, tested its correlation with directly quantified CIN and verified its ability to stratify grade 2 breast carcinomas according to good and poor clinical outcome in routine formalin-fixed, paraffin-embedded (FFPE) pathological samples. We compared the predictive power of the resulting signature to mitotic index and Ki67 expression.

## Materials and Methods

### In silico selection of CIN genes for expression analysis

All microarray data sets used in this analysis were normalized by robust multi-array average (RMA) [15]. Probe sets to represent each gene in the various signatures (CIN70 [1], NKI70 [16], 21-gene Recurrence Score (DX21) [17], intrinsic subtype [18], Ivshina's molecular grade [11], Ma's 5-gene HOXB13∶IL17BR ratio (Ma5) [19], Sotiriou's Genomic Grade Index (GGI) [20]) analyzed were selected by Jetset as described previously [21]. If no reliable probe set was found for a given gene that was excluded from further analysis. The data sets are displayed in Table S1.

### Tissue samples

Focusing on histological grade, we evaluated 185 invasive breast carcinomas consisting of 63 grade 1, 62 grade 2 and 60 grade 3 FFPE tissue samples regardless of other pathological features from the Buda MÁV Hospital (1999–2002), diagnosed and graded by a single pathologist (J.K.). Recurrence-free survival (RFS) time was defined either by loco-regional relapse or the appearance of a distant metastasis, and whichever shorter if both applicable. The study was a retrospective analysis. General written consent was obtained from all patients at time of surgery. The samples were anonymised for the study. The study was approved by the Ethical Committee of the Semmelweis University (IKEB #7/2008 and #7-1/2008).

### Patient characteristics

In line with bioinformatics, the clinicopathological properties of the selected 185 breast cancer patients were analyzed. The mean age of patients was 58.8±12.8 years (59.9±11.8 years, 59.2±13.0 years, 56.5±13.7 years in grade 1, 2 and 3 tumor groups, respectively). Among the histological types, invasive ductal carcinoma was the most common overall (65.9%), but less frequent types of cancer were also included in the analysis (8.1%): 1 papillary, 1 tubular, 1 micropapillary and 3 mucinous carcinomas in the grade 1 group; 1 micropapillary and 1 mucionous in grade 2; and 4 with medullary features, 2 metaplastic and 1 micropapillary carcinomas in grade 3 cancers. Tumor size, frequency of vascular invasion, presence of necrosis, Nottingham Prognostic Index (NPI) and number of relapses showed an increase, while ER, PgR expression and RFS decreased with higher grade (Table 1). When characterizing tumors according to immunophenotype, expression of ER, PgR and Her2 were evaluated and fluorescence in situ hybridisation (FISH) was performed to assess Her2 amplification. ER and PgR expressing tumors with lower than 20% Ki67 expression were considered as Luminal A (LumA). For ER or PgR and Her2 positive tumors and ER and PgR expressing tumors with more than 20% Ki67 index, Luminal B subtype (LumB) was assigned [22]. Her2 expressing and/or Her2 amplified and ER and PgR negative tumors were considered as the Her2 subgroup (none were treated with trastuzumab at that time). Triple negative breast cancers (TNBC) were ER, PgR, Her2 negative tumors with no Her2 amplification. The disease free survival of patients in the different grade- and immunophenotype groups were plotted on Kaplan-Meier graphs (Figure S1).

**Table 1:** Clinicopathological data of the 185 breast cancer patients included in the analysis.

Groups according to grade		1	2	3	All
**Patients (n)**		63	62	60	185
**Age – years/mean, (range)/**		59.9 (35–95)	59.2 (23–87)	56.5 (29–87)	58.8 (23–95)
**Histology (n, %)**	IDC	45 (71.4%)	37 (59.6%)	40 (66.6%)	122 (65.9%)
	ILC	4 (6.3%)	3 (4.8%)	1 (1.6%)	8 (4.3%)
	Mixed	8 (12.6%)	20 (32.2%)	12 (20.0%)	40 (21.6%)
	Other	6 (9.5%)	2 (3.2%)	7 (11.6%)	15 (8.1%)
**Tumour size - mm (mean** ± **SE)**		20.49±1.10	26.81±1.54	28.55±1.92	25.13±13.87
**Vascular invasion/n (%)/**		34 (55.5%)	46 (74.1%)	49 (81.6%)	129 (69.7%)
**Necrosis/n (%)/**		8 (12.6%)	16 (25.8%)	35 (58.3%)	59 (31.8%)
**NPI (mean** ± **SE)**		3.00±0.11	4.39±0.13	5.58±0.15	4.39±1.39
**Immunophenotype (n, %)**	Lum A	55 (87.3%)	40 (64.5%)	22 (36.6%)	117 (63.2%)
	Lum B	4 (6.3%)	8 (12.9%)	15 (25.0%)	27 (14.5%)
	HER2	0	3 (4.8%)	7 (11.6%)	10 (5.4%)
	TNBC	4 (6.3%)	11 (17.7%)	16 (26.6%)	31 (16.7%)
**Recurrence (n, %)**	Local relapse	8 (12.6%)	5 (8.1%)	7 (11.6%)	20 (10.8%)
	Distant metastasis	8 (12.6%)	21 (33.8%)	21 (35.0%)	50 (27.0%)
**RFS – months/mean (range)/**		85.9 (12–122)	75.9 (0–123)	74.1 (0–119)	78.8 (0–123)

### RNA purification and qPCR

Five to ten 5 µm thick sections were used from each case for RNA purification after assessment of cellularity on HE stained slides (minimum of 70% tumor cell content was required). RNA was extracted with Qiagen FFPE RNeasy kit according to the manufacturer's protocol (Qiagen, Venlo, The Netherlands). High Capacity RNA-to-cDNA kit was used to reverse transcribe 1000 ng of RNA (Applied Biosystems, Foster City, CA, USA). The Eppendorf epMotion 5070 robotic system was used to transfer samples and reagents to 384-well full-skirted white plates (Eppendorf, Hamburg, Germany). The qPCR was performed in duplicates with Taqman® Assays (Table S2) in Gene Expression Master Mix (all from Applied Biosystems) according to the manufacturer's protocol. The reactions were run in Roche LightCycler 480 real-time PCR system (Roche Diagnostics, Mannheim, Germany). The CIN4 signature resulting from the qPCR measurement was assessed based on the average expression of the four genes (AURKA, FOXM1, TOP2A, TPX2) normalized to the average expression of the three control genes. In order to find robust internal reference control genes for clinical evaluation of CIN4 by qPCR in formalin-fixed tumor tissue, we chose three known housekeeping genes which bare consistently low variance in the microarray profiles of all breast cancer datasets as normalizing genes: B4GALT3, SLC9A3R2 [23] and PUM1 [24].

### Flow cytometry

Flow cytometry was performed for the analysis of ploidy. Briefly, a 50 µm section was cut from all the FFPE blocks. A scroll of tissue was placed in a microcentrifuge tube and xylene was added to remove the paraffin wax. The tissue was then serially re-hydrated through 100%, 95%, 70% and 50% ethanol for 5 minutes respectively at room temperature. The tissue was washed twice with distilled water. A suspension of nuclei was made by incubating the tissue in a 0.5% pepsin solution (Sigma-Aldrich, Dorset, UK) prepared in 0.9% saline at pH 1.5. Incubation was carried out at 37°C for 30 minutes. The nuclei were washed once with PBS, stained with propidium-iodine and analyzed using the Calibur 1 FACS mashine and CellQuest software (Beckman Coulter, Buckinghamshire, UK). DNA index was assigned as follows: diploids were ‘1.0’, a tumor with a DNA index greater than 1.10 was classified as aneuploid [25].

### Statistics

The assignment of each patient into two cohorts using the CIN4 expression signature was performed in the R statistical environment (R, version 2.10.1) using the package Prediction Analysis for Microarrays (PAM, version 2.19) as described previously [26]. By performing a soft thresholding to produce a shrunken centroid, which allows the weighting and ranking of genes with high predictive potential, PAM is capable to classify the samples into two cohorts. After this assignment, Kaplan-Meier curves were used to plot the efficiency of the prediction on recurrence-free survival. Chi-squared test was performed to test relation of qPCR and FACS assigned cohorts and clinicopathological variables grouped into categorical variables. Continuous variables were compared with student's t-test. Regression models and Cox multivariate regression analysis was performed using SPSS 15.0 (SPSS Inc., Chicago, IL, USA) and R (version 2.10.1, Vienna, Austria). We used two-sided tests and set a significance level of 0.05 for accepting the test p-values.

## Results

### CIN4 signature derived from in silico ranking of CIN70 genes also reflects histological grade

In order to select a more limited set of genes that optimally reflect the CIN70 signature we retrieved expression profiles for the CIN70 signature genes from 10 publicly available breast cancer datasets [1].

The CIN70 genes were then ranked by Pearson's correlation coefficients to the CIN score (mean CIN70 expression) within these breast cancer microarray cohorts [supplementary references]. In order to derive a clinically applicable qPCR expression signature for use in FFPE tissue, containing fewer genes with equivalent information reflecting mean CIN70 expression [1], Correlation with grade was not used in selecting the PCR quantified CIN genes, those were selected purely based on their highest average correlation coefficients to tumor CIN score. These four genes (AURKA, FOXM1, TOP2A, TPX2) were termed the CIN4 signature.

For 5 of the 10 breast cancer microarray cohorts histological grade was also available. The above listed 4 genes were also highly correlated with histological grade (Pearson correlation coefficient above 0.7).

Next, we assessed the association individually on data sets between the mean-expression level of CIN4 and clinical outcome across the same 10 cohorts containing expression data from 1928 breast cancers. We observed significant discriminative power (p<0.05, for all) by CIN4 for the stratification of good from poor clinical outcome in all of the breast cancer cohorts [supplementary references]. Therefore, the expression of CIN4 appears almost as efficient at predicting cancer outcome as the extended CIN25 and CIN70 signatures. We were able to compare the performance in silico of the CIN4 signature to a number of previously published predictors of outcome such as CIN25, CIN70 [1], Ki67, Ivshina's molecular grade [11], Ma5 [19], GGI [20], DX21 [17] and NKI70 [16] in 3 datasets (GIS [11], JBI [20], JBI1 [20], [27]). CIN4 performed almost as well as CIN25. The univariate model based on which the meta analysis performed clearly suggested the HR of CIN4 is slightly better than the other predictors (Figure S2a), however, we wanted to know if CIN4 classification adds any predictive power to existing classifiers (predictorX). In another analysis, testing the additive power from CIN4 to a given predictor we determined the following model: survival = CIN4+predictorX+CIN4:predictorX. If CIN4 adds any predictive power, the benefit is represented by a significant interaction (CIN4:predictorX). However, we did find that even though CIN4 has a higher HR in the univariate model it does not add significant predictive power to other signatures (Figure S2b).

### Identification of control genes in datasets and their variability in the tissue samples

B4GALT3, SLC9A3R2 and PUM1 were previously chosen based on their low variability in gene expression datasets described previously [23], [24]. The three genes showed low variation between the investigated samples as well (Table S3).

### CIN4 stratifies outcome in grade 2 tumors in a cohort of 185 breast cancers

Expression of CIN4 was assessed in a retrospective cohort of 185 patients for which we had FFPE primary breast cancer samples available from Buda MÁV hospital, treated between 1999 and 2002 (Table 1). Kaplan-Meier curves of the pathological evaluation of Ki67 expression and mitotic index performances on prognosis are shown in Figure S3.

In order to define a threshold for CIN4 expression for distinct outcome groups, we trained the PAM algorithm using the continuous CIN4 gene expression signature to discriminate clinical outcome of 63 patients with grade 1 breast cancer compared to the poorer outcome associated with 60 grade 3 breast cancers from within this cohort of 185 patients. Using this CIN4 expression threshold that best distinguished grade 1 compared to grade 3 breast cancers, the PAM defined CIN4 score was established, and we assessed the ability of this CIN4 score to stratify cancer outcome in the remaining 62 patients with grade 2 breast cancer from this 185 patient cohort.

**Figure 1:**
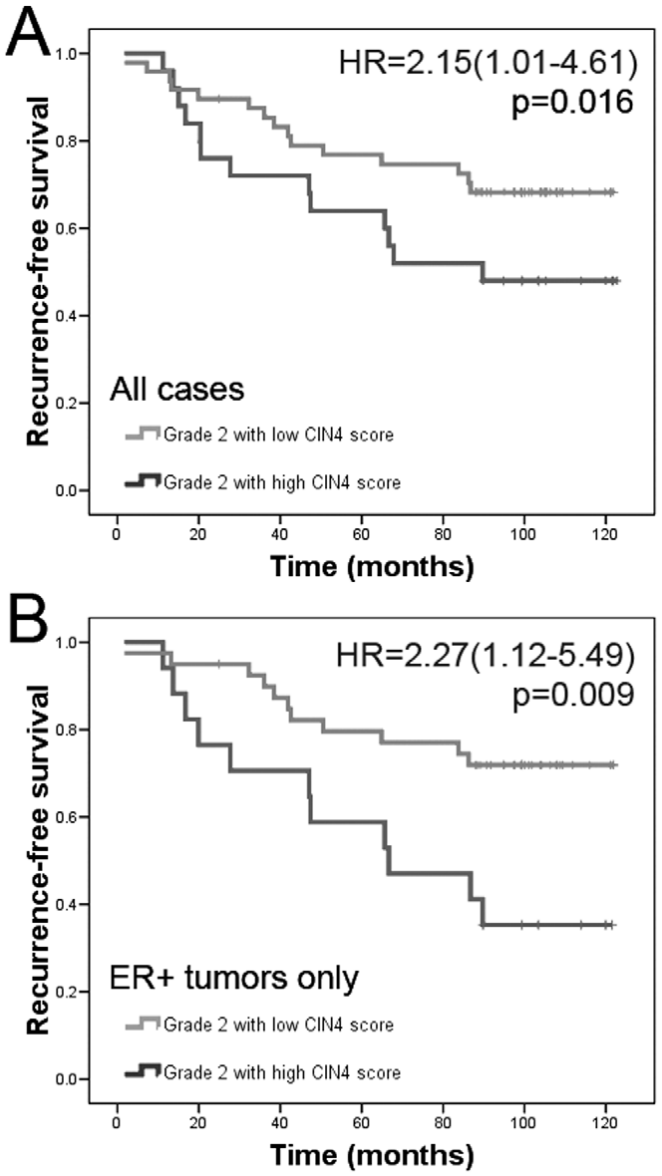
CIN4 is prognostic in the evaluated patients' samples. A) The 4-gene signature based, PAM designated CIN4 score showing discrimination between grade 2 good and poor prognostic groups plotted on Kaplan-Meier curve in the validation group (p = 0.017): 45 cases (38 ER+, 7 ER−) in low CIN4 score group and 17 cases (10 ER+, 7 ER−) in high CIN4 score group. B) The CIN4 score performing in ER+ tumors only: 38 cases in low CIN4 score group and 10 cases in high CIN4 score group (p = 0.009).

Using this threshold, the CIN4 score was able to stratify patients with grade 2 breast cancers into good (44 patients) and poor (18 patients) prognostic groups (mean, relapse-free survival: 83.8 months [95%CI: 73.6–94.2] and 69.4 months [95%CI: 55.1–90.6], respectively [p = 0.016], HR = 2.155[1.007–4.612]) (Figure 1A).

### Performance of the components of the CIN4 signature/score regardless of immunophenotype

For the identified genes that have been previously described to be of prognostic value, we have evaluated the prognostic power of AURKA, FOXM1, TOP2A, and TPX2 separately. Although, in public breast cancer datasets (grade 2, follow-up: 10 years, n = 497, split at median) all the genes showed strong predictive potential (AURKA: p = 0.047, HR = 1.33[1.00–1.78]; FOXM1: p<0.001, HR = 1.69[1.26–2.27]; TOP2A: p<0.001, HR = 1.68[1.26–2.25]; TPX2: p<0.001, HR = 1.64[1.23–2.18] for relapse-free survival (Figure S4), the data generated by qPCR was unable to distinguish prognostic subgroups for AURKA, FOXM1, and TPX2 at 10, 25, 75, 90 percentiles, median or average expression in our limited number of patients (Table S4). While TOP2A was already able to split prognostic subgroups at 25 percentile and median expression by itself (Table S4). The gain in performance is presented in the same table when considering all the four (CIN4) genes simultaneously.

### Performance of CIN4 in ER positive breast tumors

We assessed the performance of the CIN4 score in ER+/HER2− tumors classified by immunohistochemistry. This cohort was also stratified into good and poor prognostic groups with reasonable confidence (p = 0.009, HR = 2.269[1.117–5.486], Figure 1B), suggesting that the CIN4 score-defined group with poor outcome is not driven by the worse outcome of the HER2+ subgroup diagnosed and treated before the introduction of trastuzumab. Other subtypes were under-represented and comparison could not be made in the herein investigated cohort.

### CIN4 expression signature reflects aneuploidy directly quantified by FACS analysis

**Figure 2:**
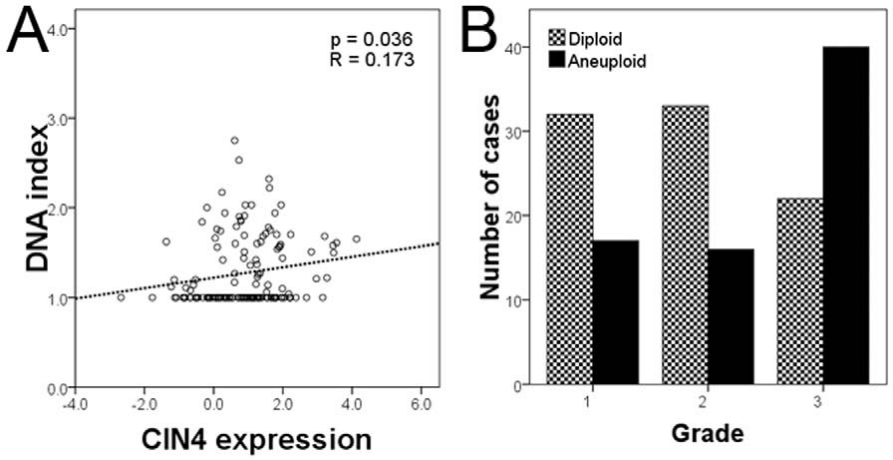
CIN4 expression correlates with ploidy. A) Regression curve showing relation of CIN4 expression signature and DNA index (p = 0.036). B) Bar graph showing numbers of diploid and aneuploid cases grouped according to histological grade: grade 3 tumors are relatively enriched in aneuploid cancers.

We assessed tumor DNA index through flow cytometry, classifying cancers into aneuploid and diploid categories according to standard threshold (threshold = 1.10 [25]). We evaluated the relationship of DNA index and the CIN4 expression by regression analysis. The variables showed a significant relationship between CIN4 expression and increasing DNA index (p = 0.036, Figure 2A). From Fig. 2A, a substantial proportion of tumors with high CIN4 scores have a normal DNA ploidy (diploid cases: 44 below, 48 above mean expression of CIN4 signature; aneuploid cases: 34 below, 50 above mean expression of CIN4 signanture). In this cohort, aneuploid tumors defined by flow cytometry were relatively enriched within the grade 3 tumor group (Figure 2B). As expected, when all breast tumors were considered, ploidy status correlated with histological grade (p = 0.004).

### CIN4 expression signature and DNA index correlates with clinicopathological variables

**Figure 3:**
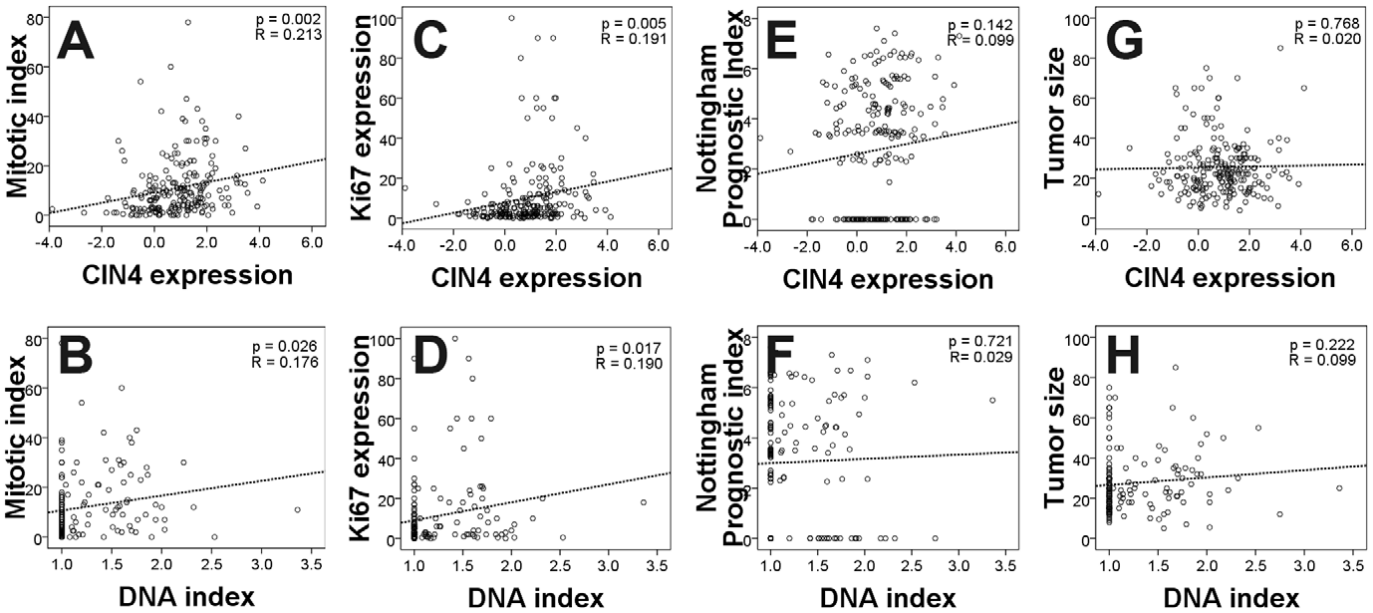
CIN4 and ploidy and their relation to pathological variables. Relation of CIN4 and DNA index to A) and B) mitotic index, C) and D) Ki67 expression, E) and F) Nottingham Prognostic Index and G) and H) tumor size displayed with regression curves (coefficients and p-values on graphs).

Using regression analysis, considering all 185 patients, the CIN4 expression signature was significantly correlated with mitotic index (p = 0.001, Figure 3A) and Ki67 expression (p = 0.005, Figure 3C), suggesting that proliferation is correlated with tumor aneuploidy (p = 0.014, p = 0.023, respectively; Figure 3B and D). The Nottingham Prognostic Index (Figure 3E and F) or tumor size (Figure 3G and H) showed a trend only towards correlation with CIN4 signature or with DNA index.

Estrogen and progesterone receptor expression as measured by IHC inversely correlated with CIN4 expression (p = 0.012, p = 0.017, respectively, Figure S5A and S5B). HER2 expression and HER2/C17 FISH counts correlated with CIN4 expression (p = 0.001, p = 0.013, respectively, Figure S5C and S5D), indicating that ER negativity and HER2 positivity are associated with higher CIN4 expression. The data derived from a qPCR assessment of CIN4 mRNA expression from FFPE breast cancers, confirms that the expression of this signature appears to be relatively enriched within ER negative and HER2 positive breast cancers (Figure S6).

### CIN4 outperforms known clinical clinicopathological parameters in multivariate outcome models

CIN4 score and clinicopathological variables were tested for their correlation in grade 2 cancers (Table S5). Cox multivariate regression analysis was then performed in grade 2 breast carcinomas: in multiple models CIN4 outperformed clinicopathological variables including hormone receptor status, tumor size, NPI, mitotic index and Ki67 index, although, when considering concordance indices the models' quality is only modest (Table 2).

**Table 2:** Multivariate regression analysis considering grade 2 breast cancers regarding the CIN4 score and clinicopathological variables.

		(1a)			(1b)			(2a)			(2b)			(3a)			(3b)	
	p-value	HR	CI	p-value	HR	CI	p-value	HR	CI	p-value	HR	CI	p-value	HR	CI	p-value	HR	CI
***HR STATUS***	0.820	0.87	0.36–3.64	0.991	0.10	0.32–3.13												
***KI67***													0.339	0.98	0.92–1.02	0.493	1.02	0.93–1.03
***LYMPH NODE STATUS***													0.816	0.96	0.65–1.39	0.914	0.98	0.70–1.47
***AGE***							0.114	1.03	0.94–1.00	0.168	1.02	0.94–1.01						
***VASCULAR INVASION***							0.714	1.20	0.31–2.18	0.799	1.13	0.34–2.27						
***NECROSIS***							0.067	0.43	0.94–5.88	0.183	0.55	0.75–4.30						
***NPI***							0.430	1.07	0.78–1.10	0.625	1.04	0.81–1.13						
***MITOTIC INDEX***	0.332	1.04	0.90–1.03	0.658	1.02	0.92–1.06												
***TUMOR SIZE***	0.847	1.00	0.98–1.03	0.903	1.00	0.98–1.03							0.690	1.01	0.97–1.03	0.919	1.00	0.97–1.02
***CIN4***	0.028	0.38	1.11–6.21				0.011	0.33	1.28–6.99				0.041	2.41	1.03–5.59			
***Observations***		63			63			64			64			63			63	
***Concordance***		0.624			0.500			0.664			0.609			0.611			0.501	
***R2***		0.073			0.005			0.131			0.046			0.070			0.010	
***Max. Possible R2***		0.950			0.950			0.953			0.953			0.950			0.950	
***Log likelihood***		−91.920			−94.155			−93.619			−96.598			−92.009			−93.973	
***Wald Test***		5.070 (df = 4)			0.280 (df = 3)			8.820 (df = 5)			3.040 (df = 4)			4.760 (df = 4)			0.560 (df = 3)	
***LR Test***		4.760 (df = 4)			0.289 (df = 3)			8.988 (df = 5)			3.028 (df = 4)			4.580 (df = 4)			0.653 (df = 3)	
***Score (Logrank) Test***		5.306 (df = 4)			0.278 (df = 3)			9.437 (df = 5)			3.052 (df = 4)			5.010 (df = 4)			0.570 (df = 3)	

Multiple variables tested in separate runs when CIN4 was included (1–3a) or excluded (1–3b) from the comparison.

## Discussion

In this study, we developed a 4 gene-based measure of CIN applicable to FFPE material demonstrating clinical utility as a fairly robust marker of breast cancer prognosis. Aneuploidy determined by FACS-based DNA index correlated with the CIN4 signature, indicating that the CIN4 signature may in fact serve as a surrogate method to determine tumor aneuploidy status.

These data derived from a qPCR assessment of CIN4 mRNA expression from FFPE breast cancers, seems to confirm our previous analysis from microarray expression datasets [5], that CIN signature expression appears to be relatively enriched within ER negative breast cancers as well.

Directly or indirectly quantifying CIN in human tumor biopsies may hold significant potential for clinical practice [2], [7], [10], [28], [29]. The aim of the current work has been to establish such a gene expression based indirect measure and validate its utility in an already existing retrospective FFPE cohort. In multivariate regression analysis the CIN4 signature confers prognostic power in comparisons across grade 2 cancers specifically. ER status and vascular invasion showed predictive power, while the CIN4 score seems to outperform mitotic index and Ki67 expression in multivariate analysis.

The CIN4 signature performed comparably to previously published gene signatures in similar cohorts. For example, the previously published Genomic Grade Index was further simplified and converted into a qPCR-based test from formalin-fixed, paraffin-embedded tissues [13], [20]. It examines the expression of 4 genes, CDC2, CDC20, KPNA2 and MYBL2 and its utility has been demonstrated to predict outcome following tamoxifen treatment. A 5-gene assay was also developed quantifying the expression of CHDH, HOXB13, IL17BR, MIB1 and MKI67 to identify a subgroup of early stage estrogen receptor–positive breast cancer patients with very poor outcome despite endocrine therapy [19]. Considering the previously published correlation between Genomic Grade Index and CIN [1], [20], it is reassuring to see that both CIN4 and the 4-gene Genomic Grade Index signature yield similar results [13], [30], indicating that replacing histological grade 2 with gene expression based low and high risk cases could be considered in prospective studies.

Interestingly, in the study we identified a group of samples with a diploid DNA index which have a wide range of CIN scores. There might be several explanations behind this phenomenon including tumor heterogeneity or the presence of yet unknown other compensatory mechanisms [31]. Clearly, a substantial proportion of tumors with high CIN4 scores have a normal DNA ploidy, indicating that the CIN4 score likely reflects factors in addition to DNA copy number. In these cases, it is possible that highly proliferative cells, although with relatively stable genomes, may have high levels of the genes comprising the signature

While establishing a qPCR-based simple test to improve histological classification is an important clinical goal, – following further validation – the CIN4 signature as a quantifier of CIN is expected to serve as a potential marker of other clinical characteristics as well. Most prominently, the putative role of CIN in determining taxane response would suggest that the predictive role of CIN4 should be tested in the neoadjuvant setting when comparing response to therapy with or without taxanes in the treatment of hormone receptor negative breast cancer.

## Supporting Information

Figure S1 **Kaplan-Meier plot showing the disease-free survival of the different grade and immunophenotype groups in the validation cohort, respectively.**
(TIF)

Figure S2 **In silico comparison of the performance of CIN4 vs. CIN25, CIN70, Ki67, genetic grade, HOXB13:IL17BR index, Genomic Grade Index, the 21-gene recurrence score and NKI70 in the GIS, JBI and JBI1 datasets** [11], [20], [27] **(A), and assessing the additive power of CIN4 (B).**
(PDF)

Figure S3 **Kaplan-Meier curves of Ki67 and mitotic index performances in the tissue samples of grade 2 breast tumors.**
(TIF)

Figure S4 **Individual prognostic performance of AURKA, FOXM1, TOP2A and TPX2 in breast cancer datasets as compared to CIN4 signature (groups split at median expression).**
(PDF)

Figure S5 **Correlation of CIN4 and markers used in the determination of immunophenotype.** CIN4 and A) ER, B) PR, and C) HER2 expression, and D) HER2/chromosome 17 score determined by FISH.
(TIF)

Figure S6 **CIN4 expression in ER-negative and ER-positive tumors.**
(TIFF)

Table S1 **The public datasets analysed in the study and their supplementary references.**
(XLS)

Table S2 **The probes used for the qPCR evaluation in the study.**
(XLS)

Table S3 **Expression of control genes in the breast cancer tissue samples according to grade.**
(XLS)

Table S4 **Performance of the individual components of CIN4 in tissue samples of grade 2 breast tumors.**
(XLS)

Table S5 **Correlation of the clinicopathological/prognostic variables with each other in grade 2 breast tumours only.** Pearson correlation coefficients displayed, significant correlation: gray highlight.
(XLS)

## References

[1] Carter SL, Eklund AC, Kohane IS, Harris LN, Szallasi Z (2006) A signature of chromosomal instability inferred from gene expression profiles predicts clinical outcome in multiple human cancers. Nat Genet 38: 1043–1048. 16921376 10.1038/ng1861

[2] Habermann JK, Doering J, Hautaniemi S, Roblick UJ, Bündgen NK, et al (2009) The gene expression signature of genomic instability in breast cancer is an independent predictor of clinical outcome. Int J Cancer 124: 1552–1564. 19101988 10.1002/ijc.24017 PMC2707256

[3] Swanton C, Nicke B, Schuett M, Eklund AC, Ng C, et al (2009) Chromosomal instability determines taxane response. Proc Natl Acad Sci U S A 106: 8671–8676. 19458043 10.1073/pnas.0811835106 PMC2688979

[4] Lee AJX, Endesfelder D, Rowan AJ, Walther A, Birkbak NJ, et al (2011) Chromosomal Instability Confers Intrinsic Multidrug Resistance. Cancer Research 71: 1858–1870. 21363922 10.1158/0008-5472.CAN-10-3604 PMC3059493

[5] Birkbak NJ, Eklund AC, Li Q, McClelland SE, Endesfelder D, et al (2011) Paradoxical Relationship between Chromosomal Instability and Survival Outcome in Cancer. Cancer Res 71: 3447–3452. 21270108 10.1158/0008-5472.CAN-10-3667 PMC3096721

[6] Roylance R, Endesfelder D, Gorman P, Burrell RA, Sander J, et al (2011) Relationship of extreme chromosomal instability with long-term survival in a retrospective analysis of primary breast cancer. Cancer Epidemiol Biomarkers Prev 20: 2183–2194. 21784954 10.1158/1055-9965.EPI-11-0343 PMC3199437

[7] Lengauer C, Kinzler KW, Vogelstein B (1998) Genetic instabilities in human cancers. Nature 396: 643–649. 9872311 10.1038/25292

[8] Nakamura H, Saji H, Idiris A, Kawasaki N, Hosaka M, et al (2003) Chromosomal Instability Detected by Fluorescence in Situ Hybridization in Surgical Specimens of Non-Small Cell Lung Cancer Is Associated with Poor Survival. Clin Cancer Res 9: 2294–2299. 12796398

[9] Fiegler H, Geigl JB, Langer S, Rigler D, Porter K, et al (2007) High resolution array-CGH analysis of single cells. Nucl Acids Res 35: e15. 17178751 10.1093/nar/gkl1030 PMC1807964

[10] Ellsworth R, Hooke J, Love B, Kane J, Patney H, et al (2008) Correlation of levels and patterns of genomic instability with histological grading of invasive breast tumors. Breast Cancer Research and Treatment 107: 259–265. 17351743 10.1007/s10549-007-9547-2

[11] Ivshina AV, George J, Senko O, Mow B, Putti TC, et al (2006) Genetic reclassification of histologic grade delineates new clinical subtypes of breast cancer. Cancer Res 66: 10292–10301. 17079448 10.1158/0008-5472.CAN-05-4414

[12] Dalton LW, Pinder SE, Elston CE, Ellis IO, Page DL, et al (2000) Histologic Grading of Breast Cancer: Linkage of Patient Outcome with Level of Pathologist Agreement. Mod Pathol 13: 730–735. 10912931 10.1038/modpathol.3880126

[13] Toussaint J, Sieuwerts A, Haibe-Kains B, Desmedt C, Rouas G, et al (2009) Improvement of the clinical applicability of the Genomic Grade Index through a qRT-PCR test performed on frozen and formalin-fixed paraffin-embedded tissues. BMC Genomics 10: 424. 19744330 10.1186/1471-2164-10-424 PMC2756282

[14] Reis-Filho JS, Tutt ANJ (2008) Triple negative tumours: a critical review. Histopathology 52: 108–118. 18171422 10.1111/j.1365-2559.2007.02889.x

[15] Irizarry RA, Hobbs B, Collin F, Beazer-Barclay YD, Antonellis KJ, et al (2003) Exploration, normalization, and summaries of high density oligonucleotide array probe level data. Biostatistics 4: 249–264. 12925520 10.1093/biostatistics/4.2.249

[16] Van de Vijver MJ, He YD, van't Veer LJ, Dai H, Hart AAM, et al (2002) A gene-expression signature as a predictor of survival in breast cancer. N Engl J Med 347: 1999–2009. 12490681 10.1056/NEJMoa021967

[17] Paik S, Shak S, Tang G, Kim C, Baker J, et al (2004) A multigene assay to predict recurrence of tamoxifen-treated, node-negative breast cancer. N Engl J Med 351: 2817–2826. 15591335 10.1056/NEJMoa041588

[18] Perou CM, Sorlie T, Eisen MB, van de Rijn M, Jeffrey SS, et al (2000) Molecular portraits of human breast tumours. Nature 406: 747–752. 10963602 10.1038/35021093

[19] Ma XJ, Salunga R, Dahiya S, Wang W, Carney E, et al (2008) A five-gene molecular grade index and HOXB13:IL17BR are complementary prognostic factors in early stage breast cancer. Clin Cancer Res 14: 2601–2608. 18451222 10.1158/1078-0432.CCR-07-5026

[20] Sotiriou C, Wirapati P, Loi S, Harris A, Fox S, et al (2006) Gene expression profiling in breast cancer: understanding the molecular basis of histologic grade to improve prognosis. J Natl Cancer Inst 98: 262–272. 16478745 10.1093/jnci/djj052

[21] Li Q, Birkbak NJ, Gyorffy B, Szallasi Z, Eklund AC (2011) Jetset: selecting the optimal microarray probe set to represent a gene. BMC Bioinformatics 12: 474. 22172014 10.1186/1471-2105-12-474 PMC3266307

[22] Colleoni M, Rotmensz N, Peruzzotti G, Maisonneuve P, Viale G, et al (2004) Minimal and small size invasive breast cancer with no axillary lymph node involvement: the need for tailored adjuvant therapies. Ann Oncol 15: 1633–1639. 15520064 10.1093/annonc/mdh434

[23] Eisenberg E, Levanon EY (2003) Human housekeeping genes are compact. Trends in Genetics 19: 362–365. 12850439 10.1016/S0168-9525(03)00140-9

[24] Szabo A, Perou C, Karaca M, Perreard L, Quackenbush J, et al (2004) Statistical modeling for selecting housekeeper genes. Genome Biol 5: R59. 15287981 10.1186/gb-2004-5-8-r59 PMC507884

[25] Chassevent As, Jourdan M-L, Romain S, Descotes Fo, Colonna M, et al (2001) S-Phase Fraction and DNA Ploidy in 633 T1T2 Breast Cancers. Clin Cancer Res 7: 909–917. 11309341

[26] Tibshirani R, Hastie T, Narasimhan B, Chu G (2002) Diagnosis of multiple cancer types by shrunken centroids of gene expression. Proc Natl Acad Sci U S A 99: 6567–6572. 12011421 10.1073/pnas.082099299 PMC124443

[27] Loi S, Haibe-Kains B, Desmedt C, Lallemand F, Tutt AM, et al (2007) Definition of clinically distinct molecular subtypes in estrogen receptor-positive breast carcinomas through genomic grade. J Clin Oncol 25: 1239–1246. 17401012 10.1200/JCO.2006.07.1522

[28] McClelland SE, Burrell RA, Swanton C (2009) Chromosomal instability: A composite phenotype that influences sensitivity to chemotherapy. Cell Cycle 8: 3262–3266. 19806022 10.4161/cc.8.20.9690

[29] Bakhoum SF, Danilova OV, Kaur P, Levy NB, Compton DA (2011) Chromosomal Instability Substantiates Poor Prognosis in Patients with Diffuse Large B-cell Lymphoma. Clin Cancer Res 17: 7704–7711. 22184286 10.1158/1078-0432.CCR-11-2049 PMC3244806

[30] Heselmeyer-Haddad K, Chaudhri N, Stoltzfus P, Cheng JC, Wilber K, et al (2002) Detection of Chromosomal Aneuploidies and Gene Copy Number Changes in Fine Needle Aspirates Is a Specific, Sensitive, and Objective Genetic Test for the Diagnosis of Breast Cancer. Cancer Res 62: 2365–2369. 11956098

[31] Gerlinger M, Rowan AJ, Horswell S, Larkin J, Endesfelder D, et al (2012) Intratumor Heterogeneity and Branched Evolution Revealed by Multiregion Sequencing. N Engl J Med 366: 883–892. 22397650 10.1056/NEJMoa1113205 PMC4878653

