# Supplementary figures and images for: The CIN4 Chromosomal Instability qPCR Classifier Defines Tumor Aneuploidy and Stratifies Outcome in Grade 2 Breast Cancer
Source: PLoS One. 2013 Feb 26;8(2):e56707. doi: 10.1371/journal.pone.0056707 (PMC3582639; doi:10.1371/journal.pone.0056707)

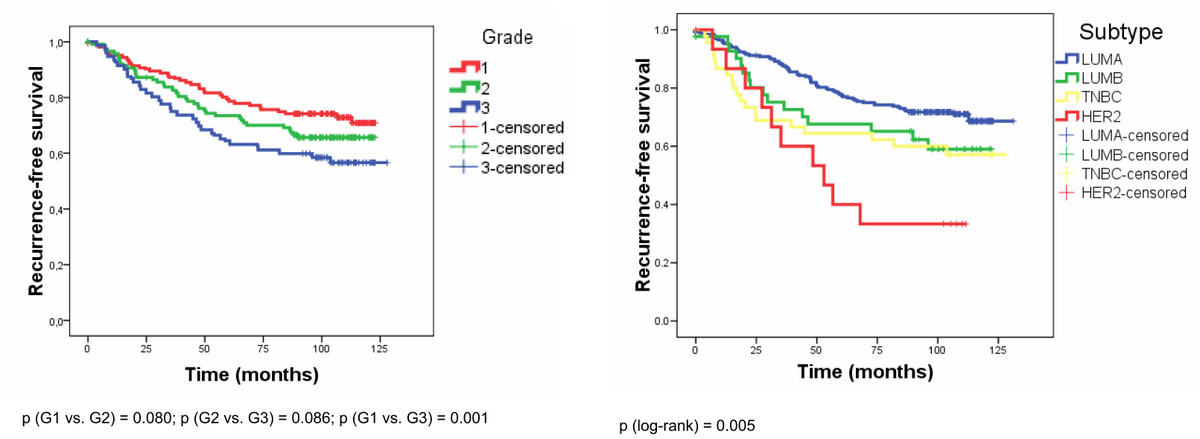

Supplement: Figure S1 — Kaplan-Meier plot showing the disease-free survival of the different grade and immunophenotype groups in the validation cohort, respectively. (TIF) [file pone.0056707.s001.tif]

CIN4

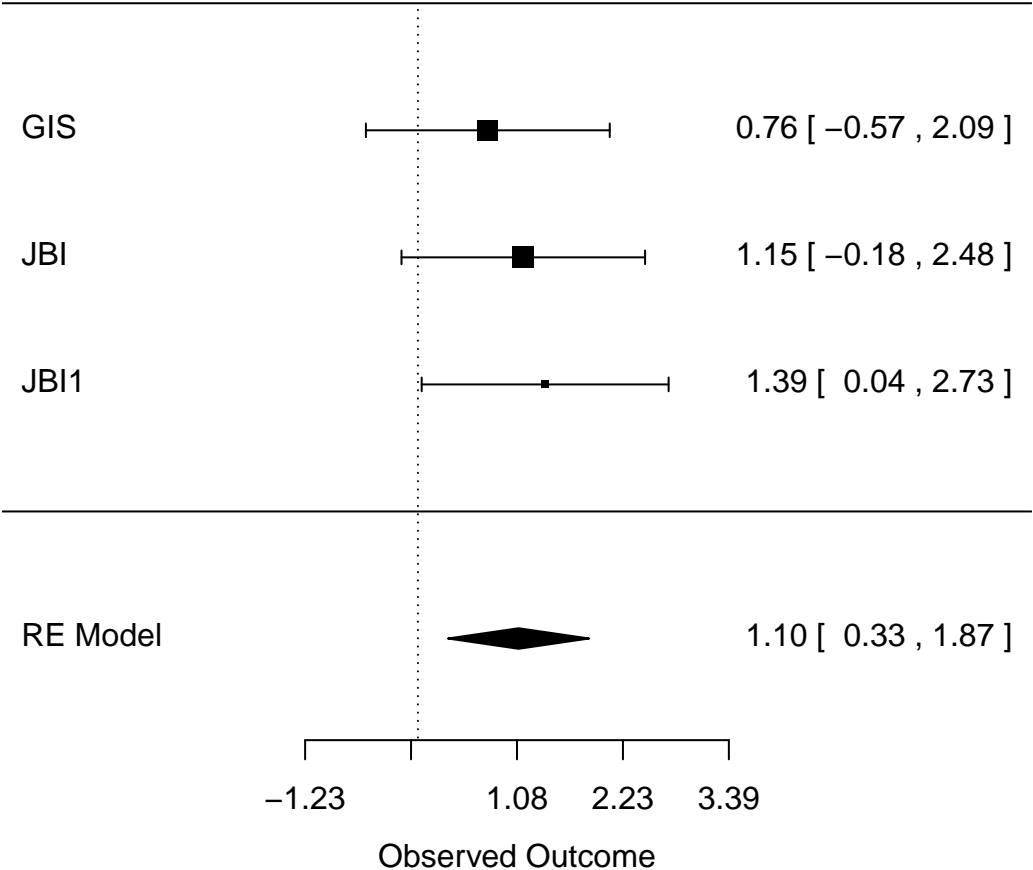

CIN25

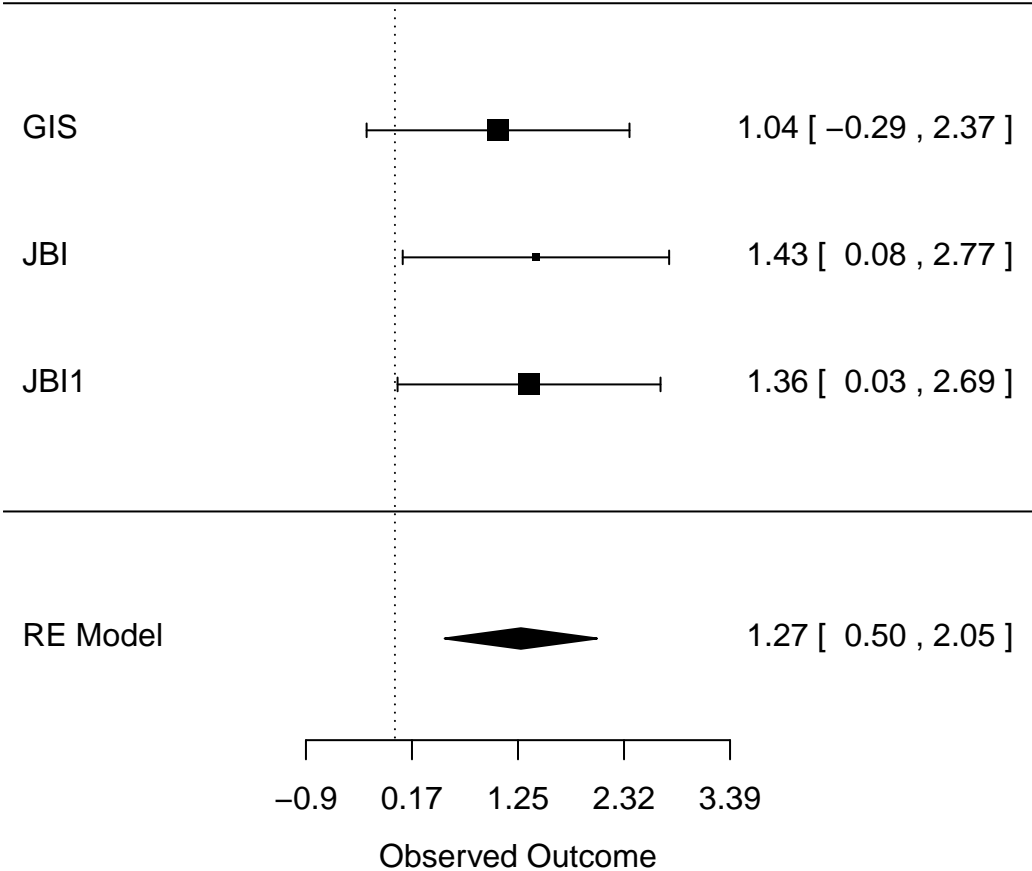

CIN70

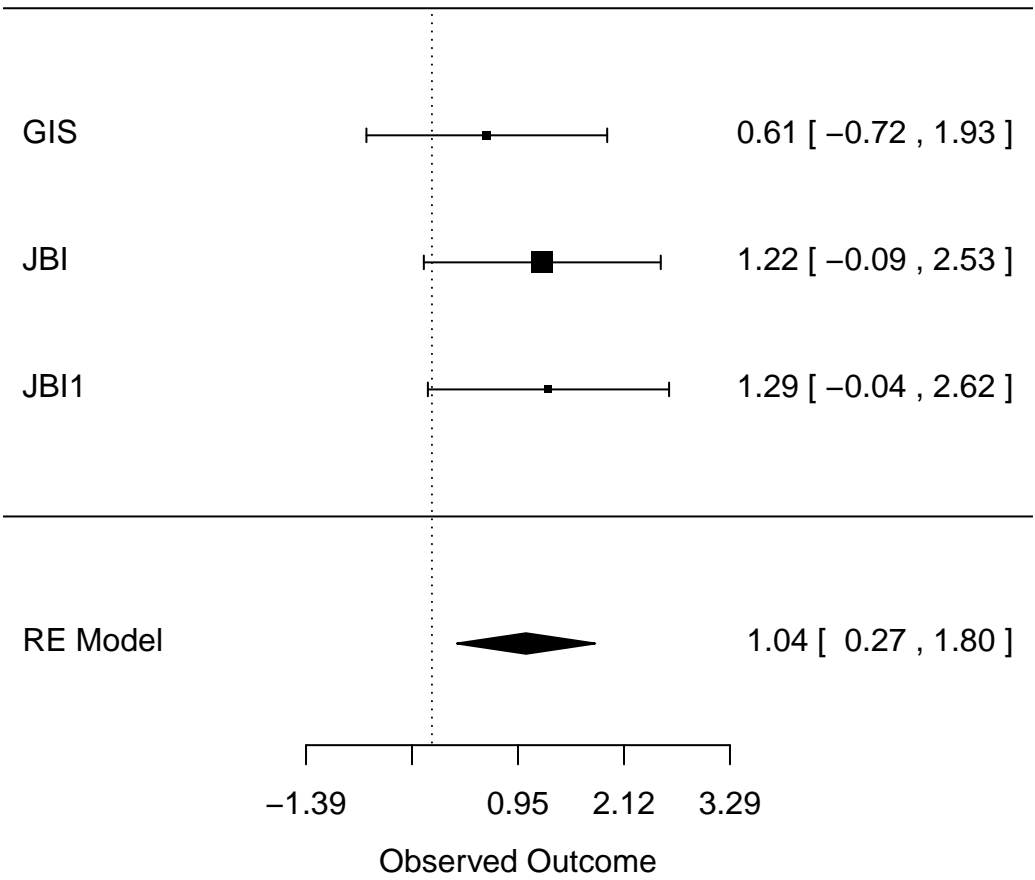

Ki67

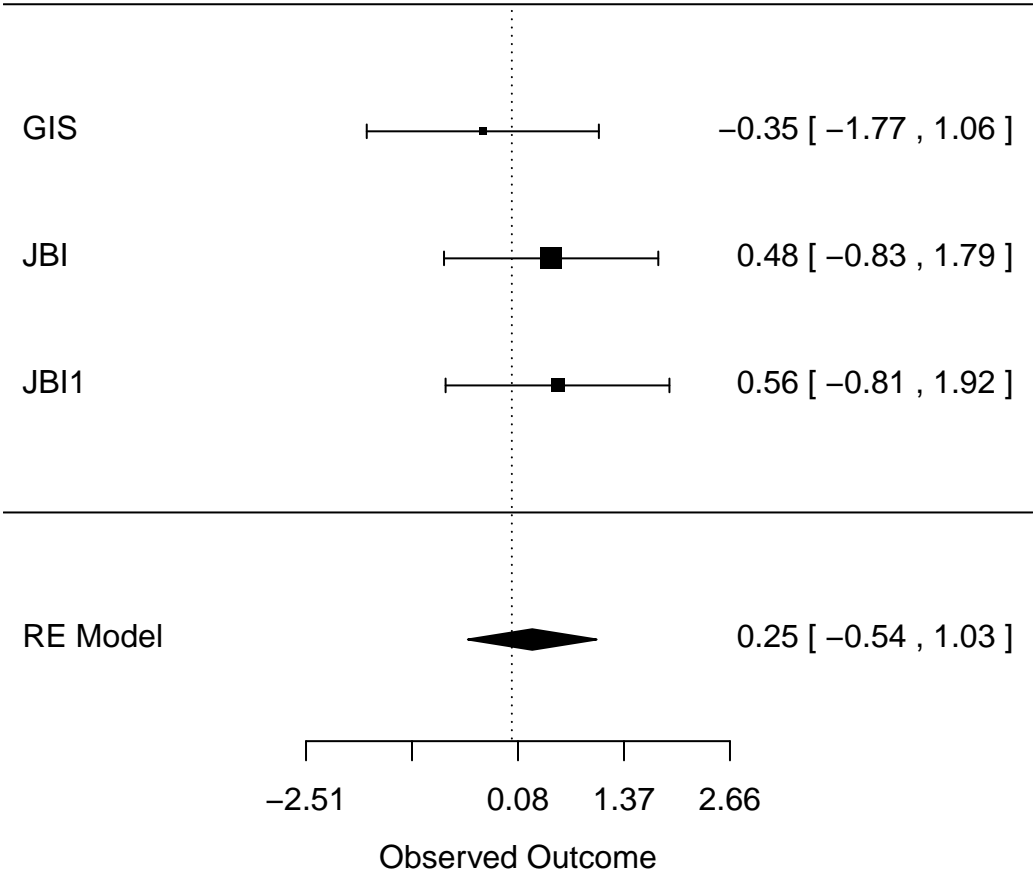

Ivshina

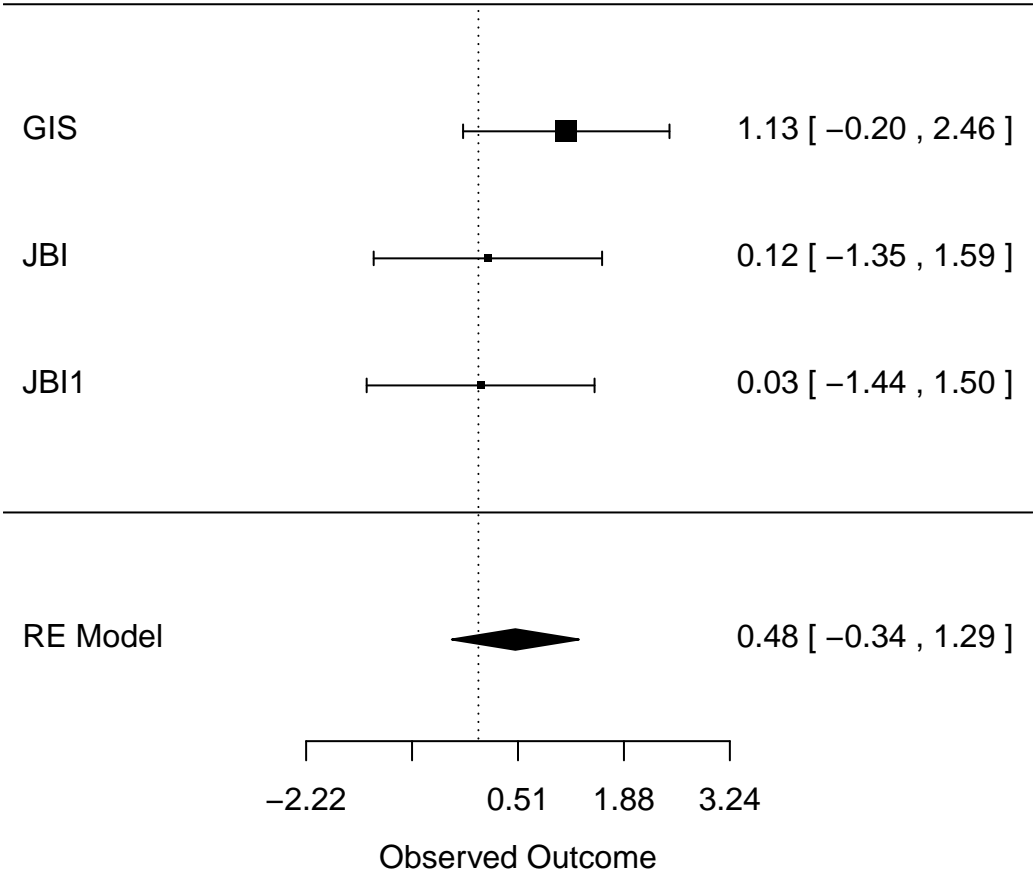

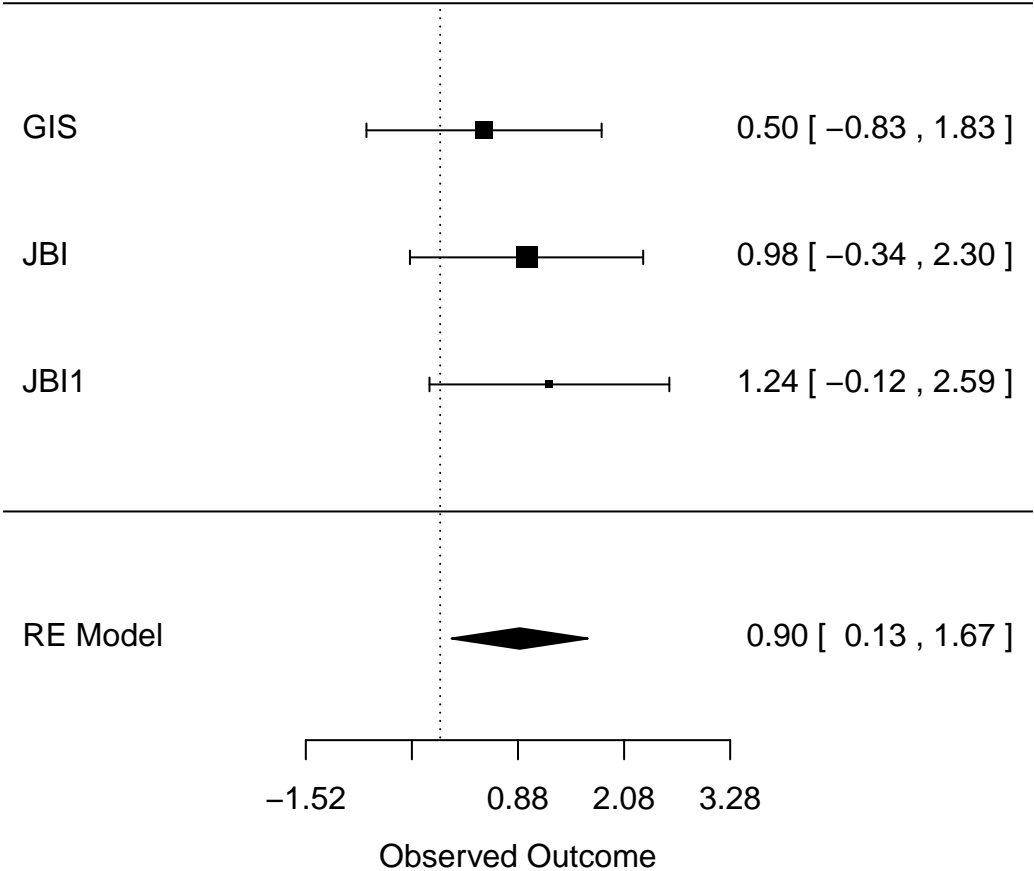

GGI

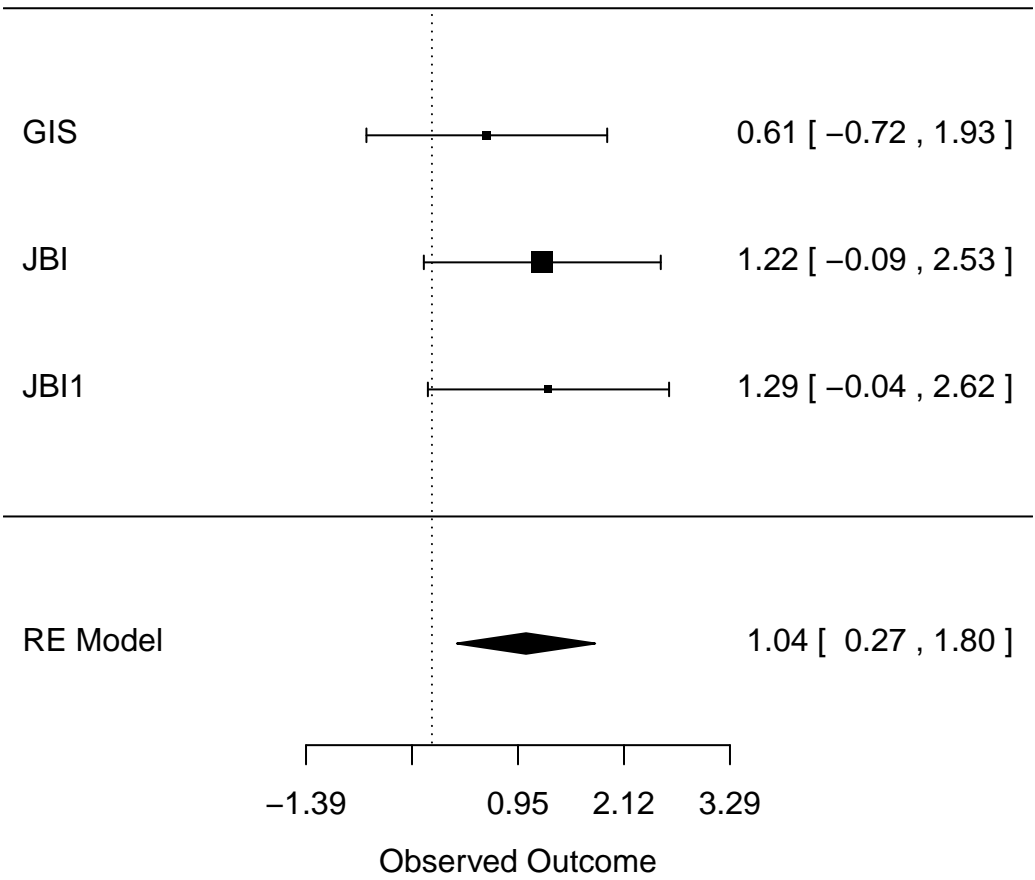

**DX21**

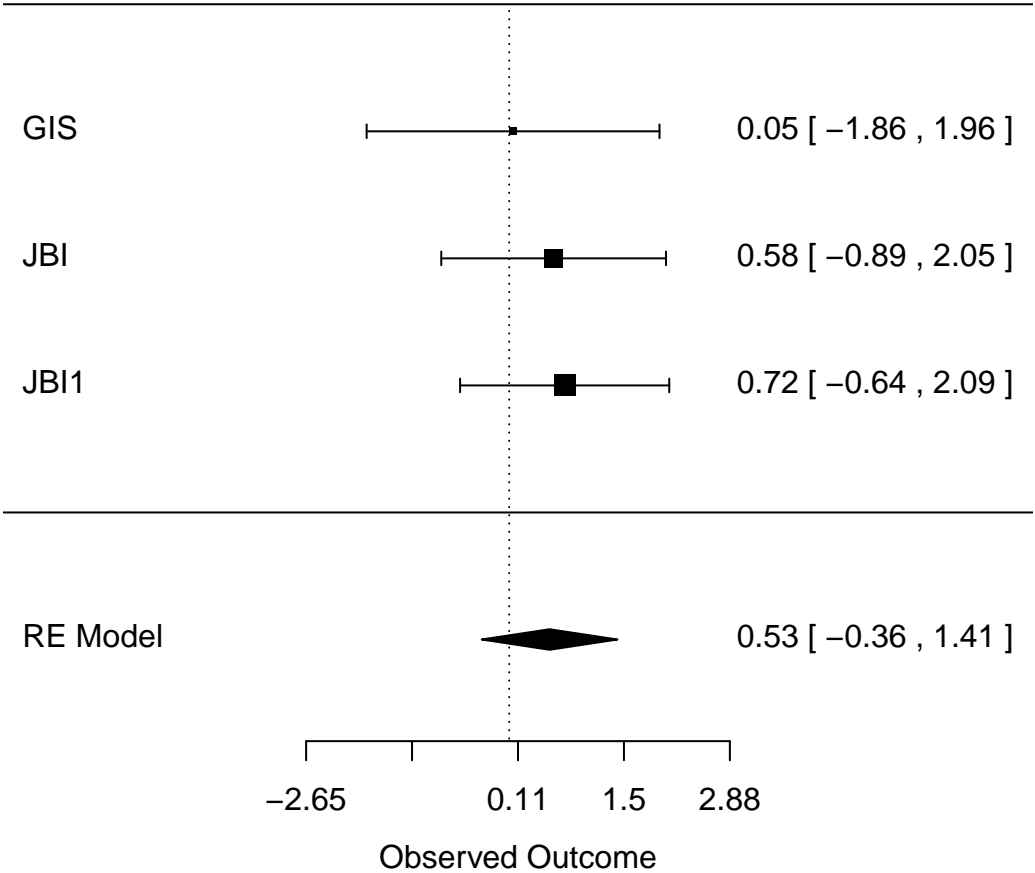

NKI70

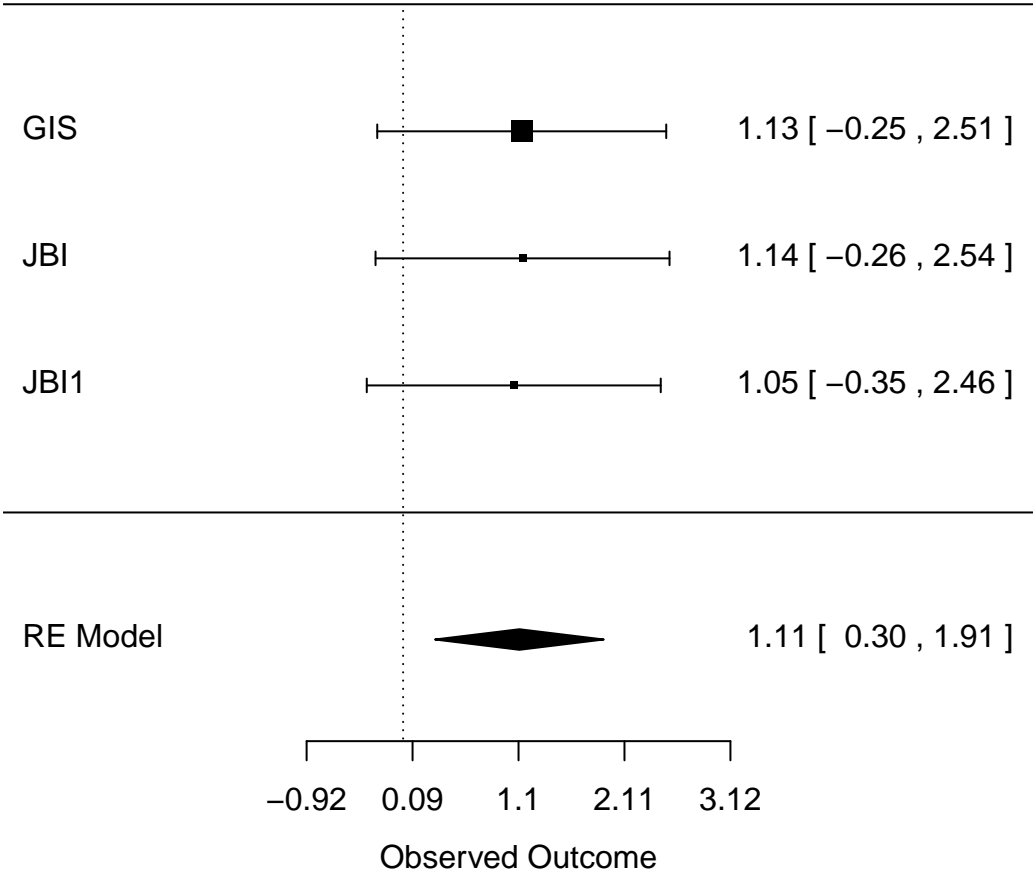

CIN25

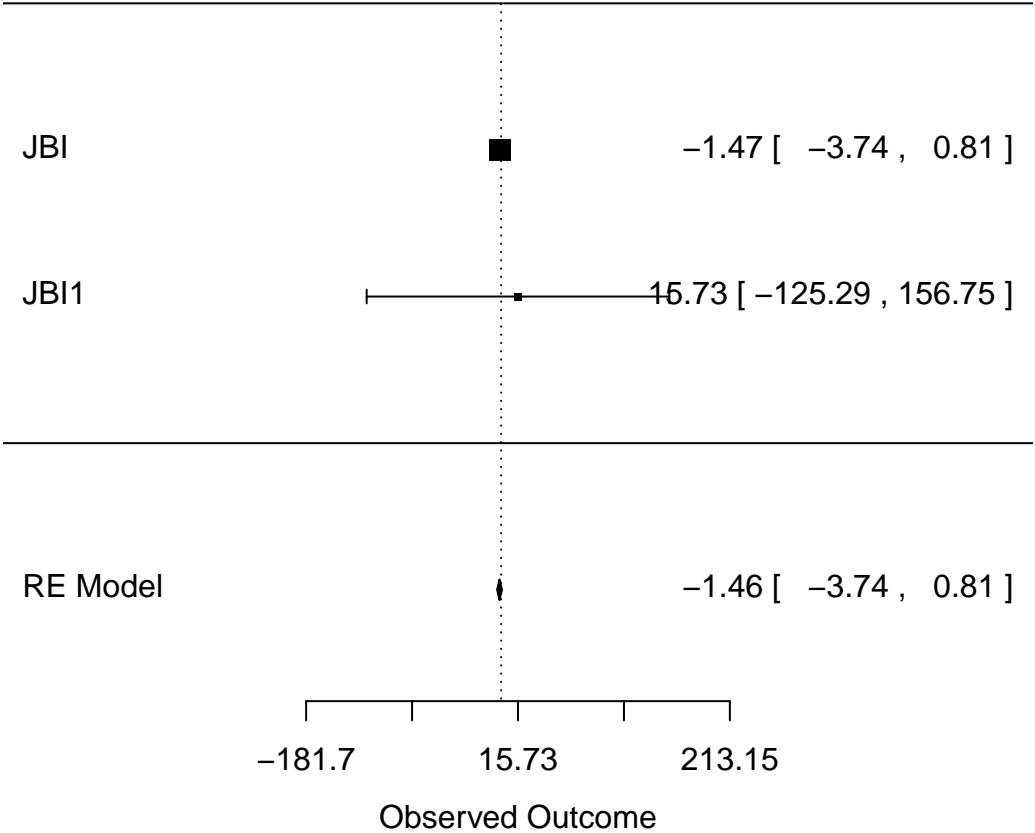

CIN61

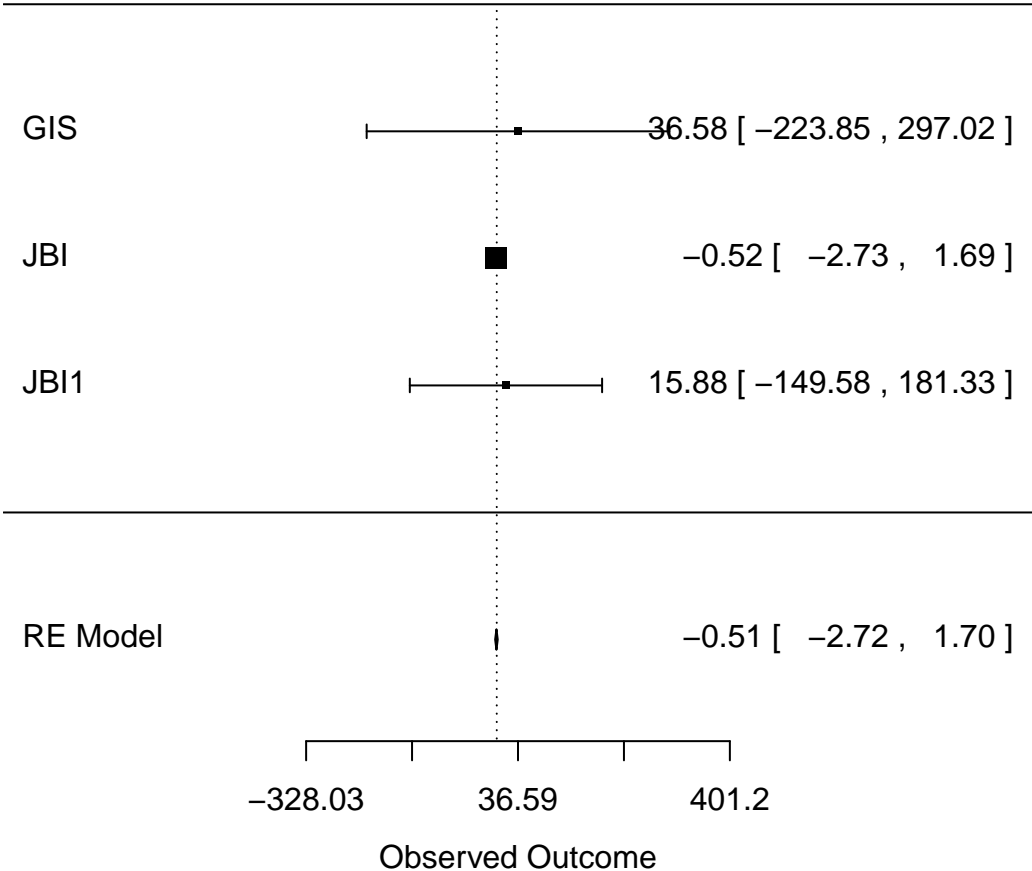

CIN70

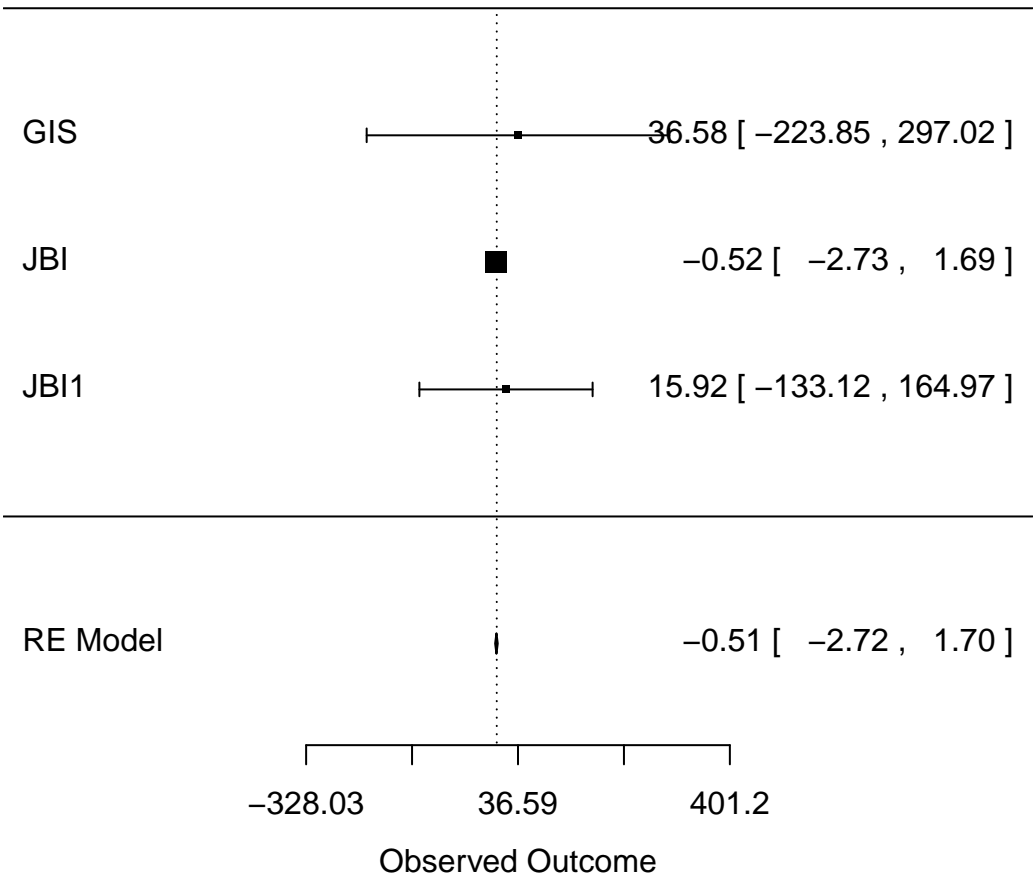

Ki67

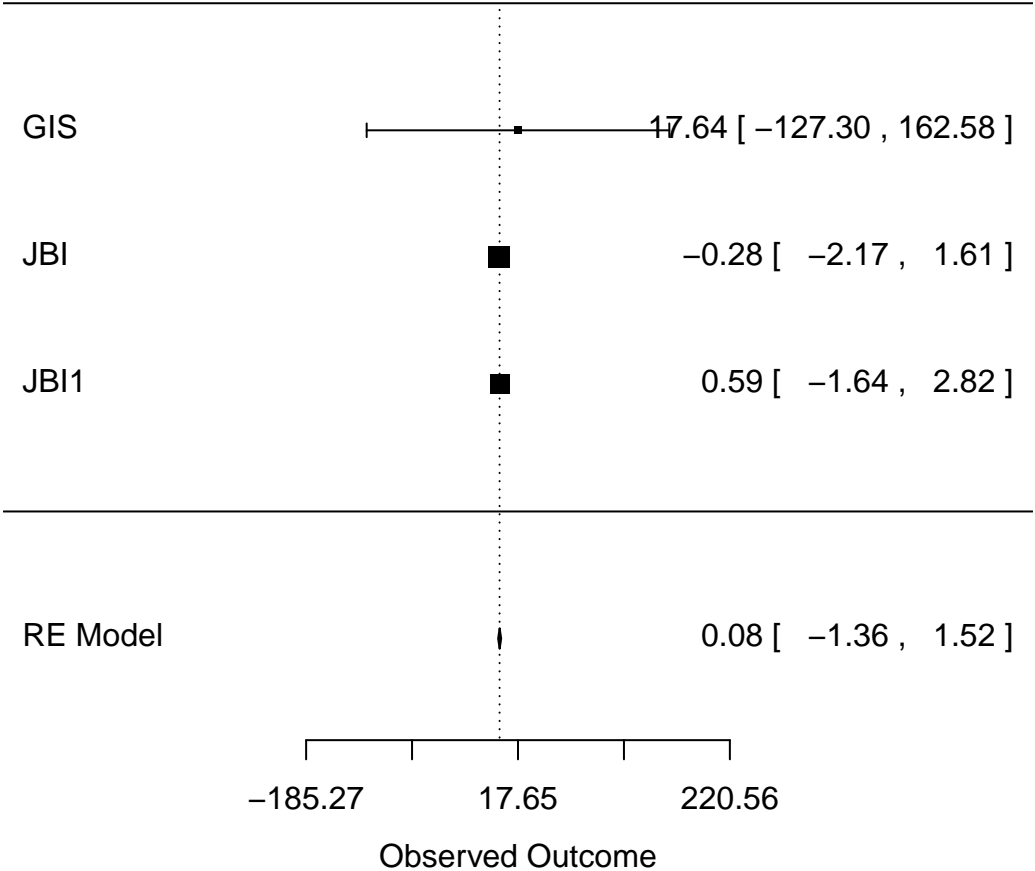

Ivshina

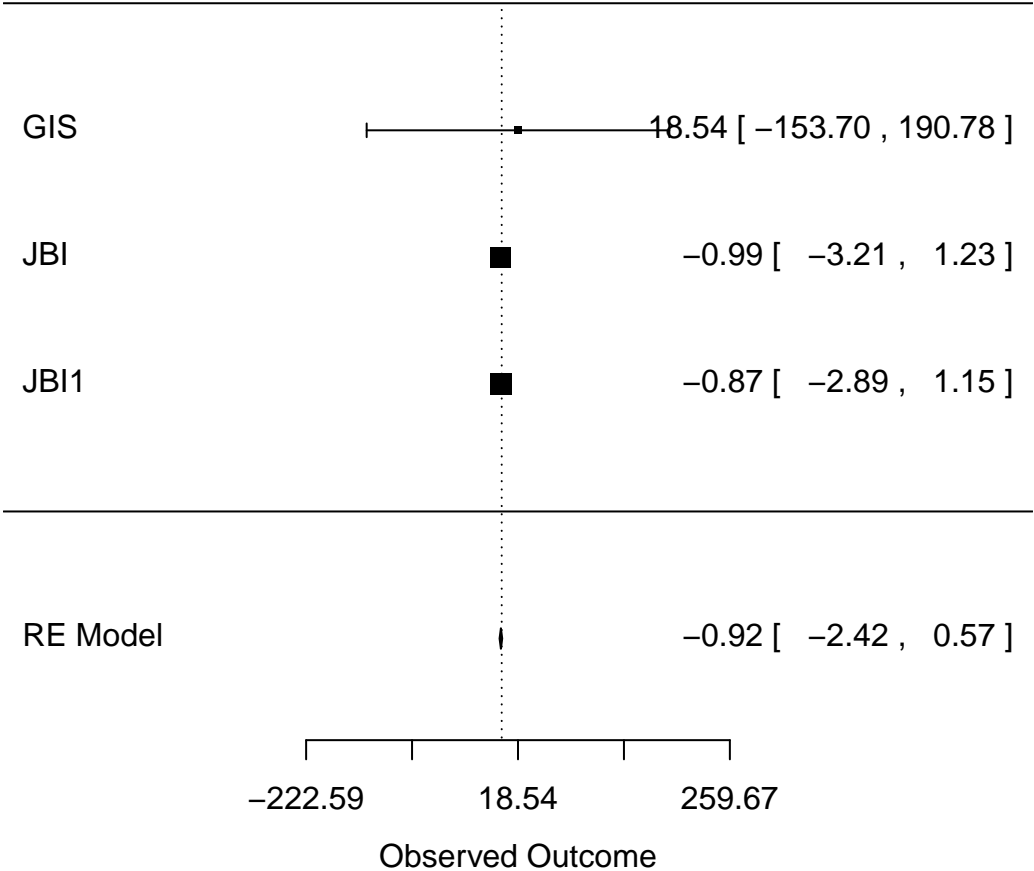

Ma

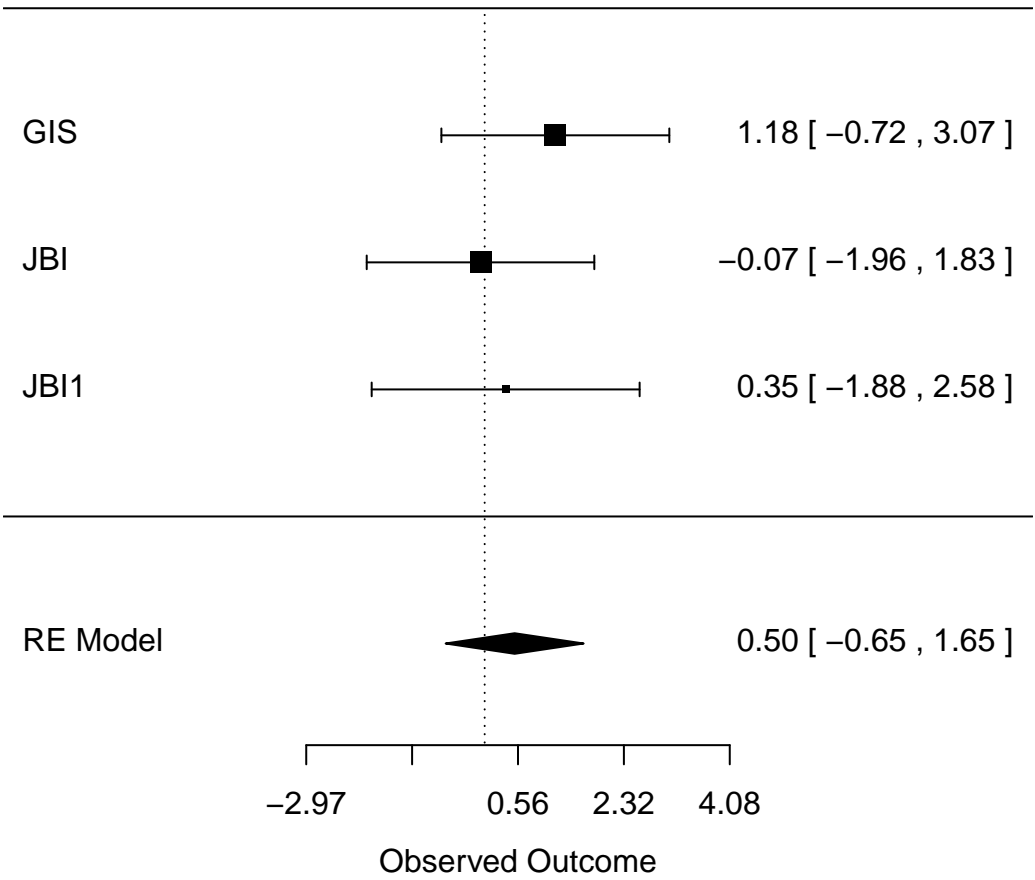

Sotiriou

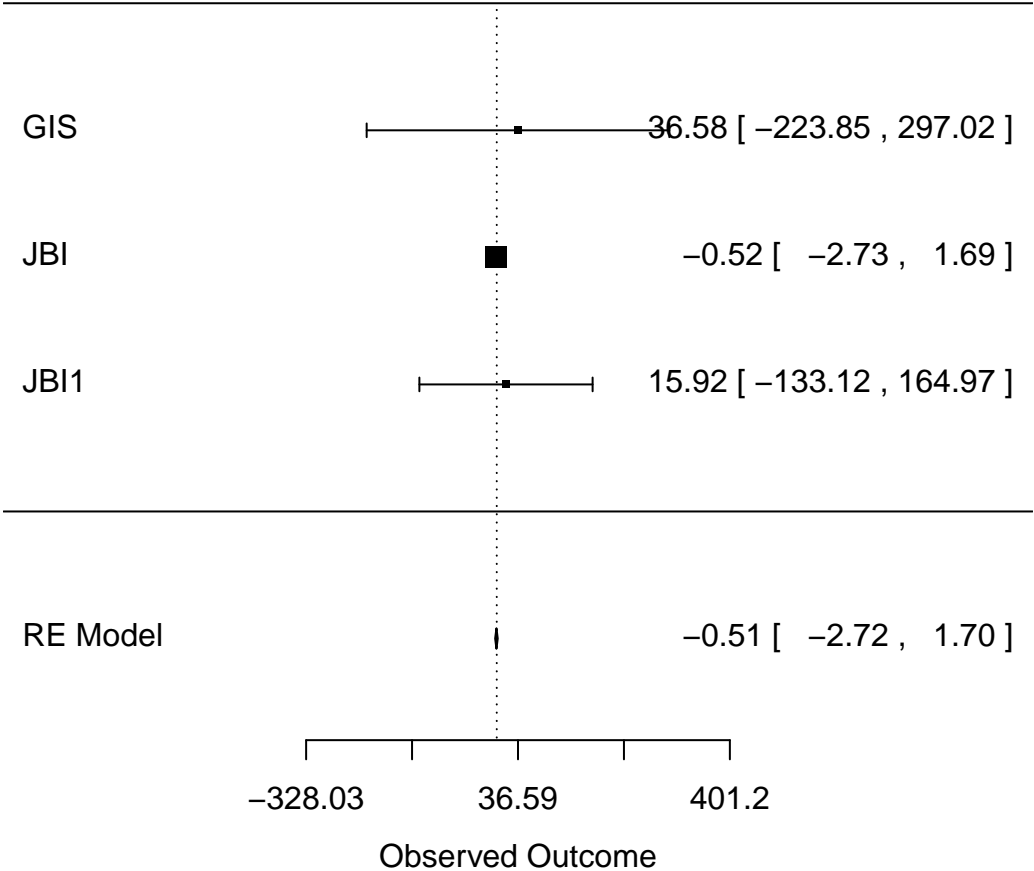

**DX21**

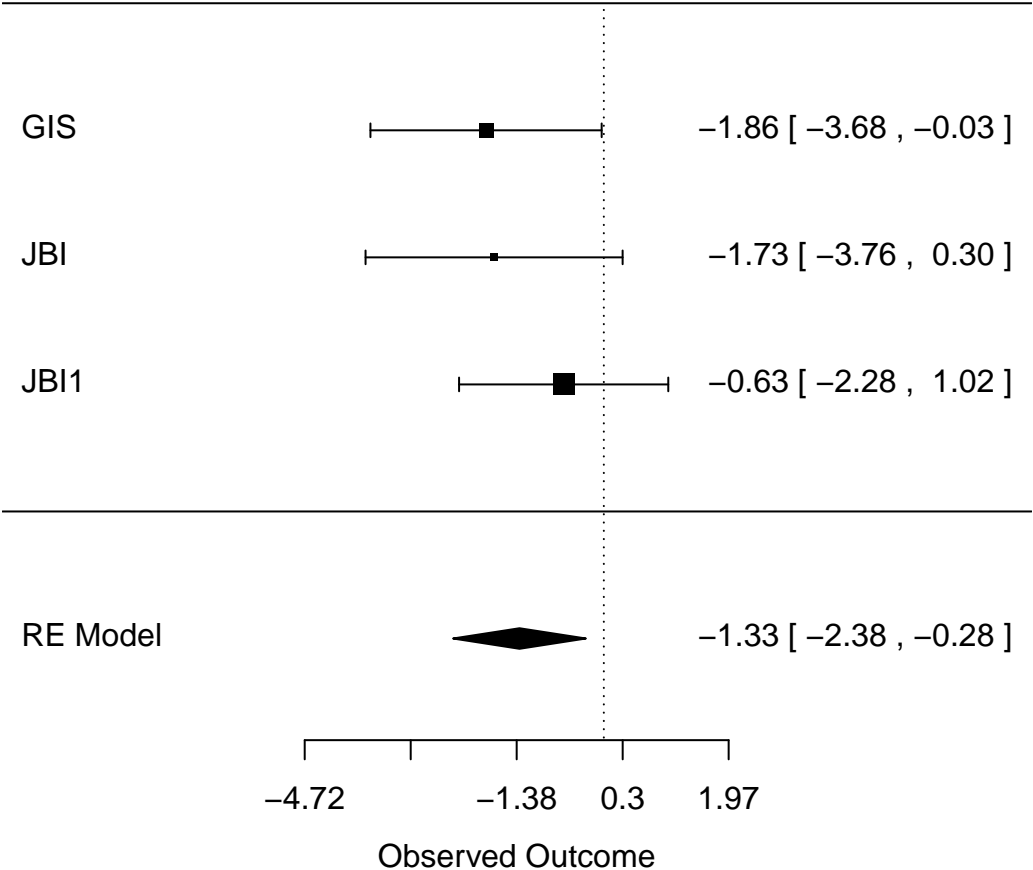

NKI70

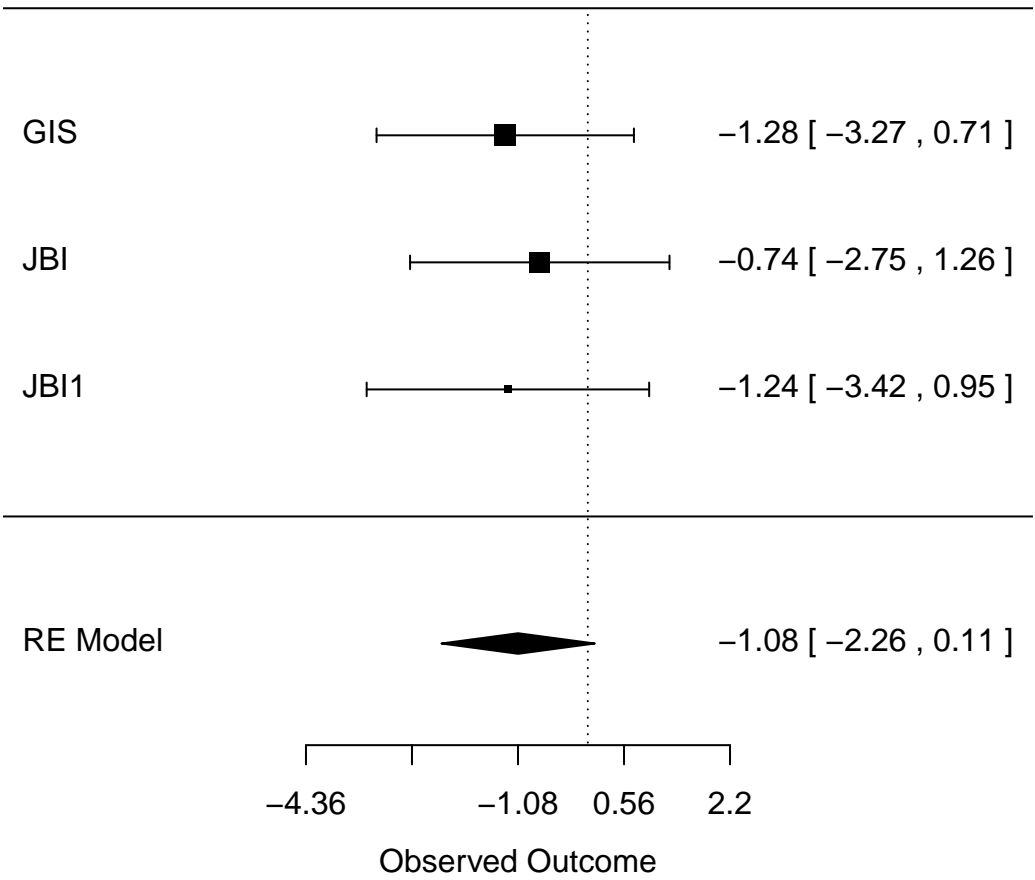

Supplement: Figure S2 — In silico comparison of the performance of CIN4 vs. CIN25, CIN70, Ki67, genetic grade, HOXB13:IL17BR index, Genomic Grade Index, the 21-gene recurrence score and NKI70 in the GIS, JBI and JBI1 datasets [11], [20], [27] (A), and assessing the additive power of CIN4 (B). (PDF) [file pone.0056707.s002.pdf]

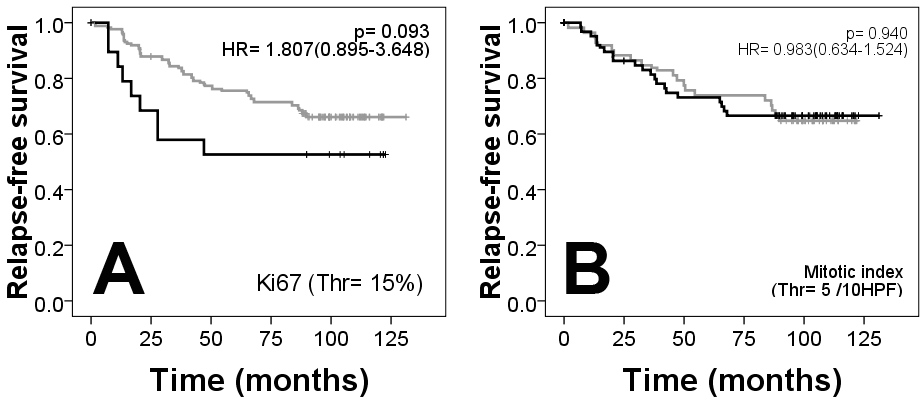

Supplement: Figure S3 — Kaplan-Meier curves of Ki67 and mitotic index performances in the tissue samples of grade 2 breast tumors. (TIF) [file pone.0056707.s003.tif]

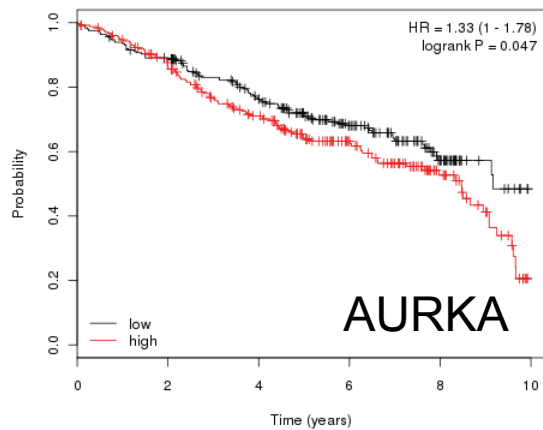

| number at risk |     |     |     |    |   |
|----------------|-----|-----|-----|----|---|
| 248            | 218 | 166 | 103 | 40 | 0 |
| 249            | 206 | 146 | 90  | 35 | 0 |

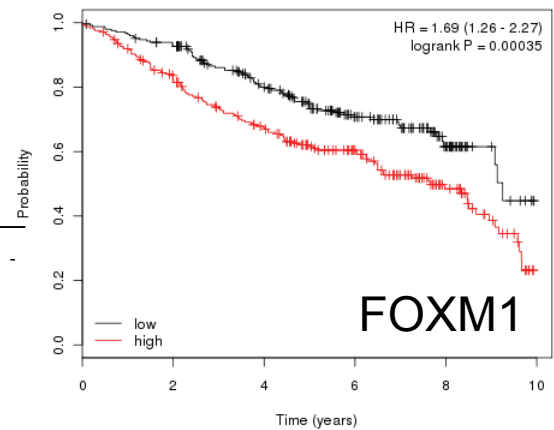

| number at risk |     |     |    |    |   |
|----------------|-----|-----|----|----|---|
| 248            | 227 | 168 | 98 | 37 | 0 |
| 249            | 197 | 144 | 95 | 38 | 0 |

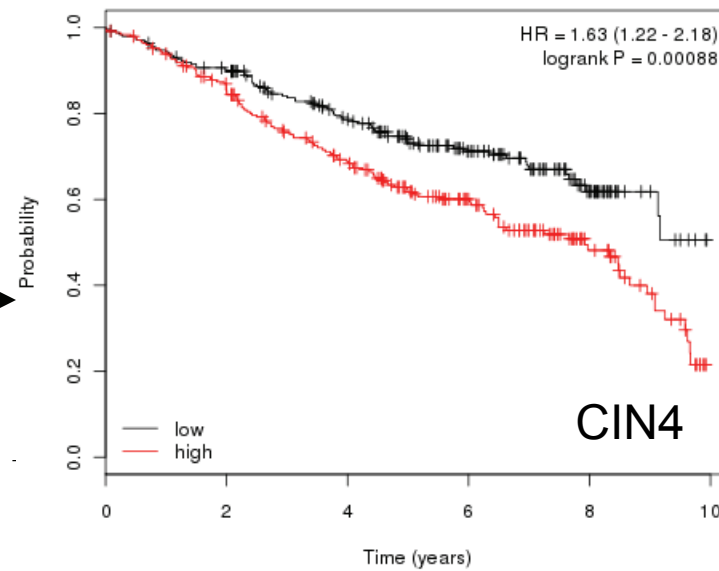

| number at risk |     |     |     |    |   |
|----------------|-----|-----|-----|----|---|
| 248            | 218 | 167 | 104 | 39 | 0 |
| 249            | 206 | 145 | 89  | 36 | 0 |

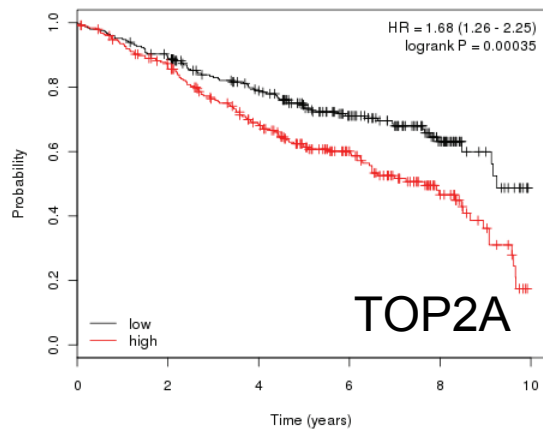

| number at risk |     |     |     |    |   |
|----------------|-----|-----|-----|----|---|
| 248            | 218 | 171 | 108 | 43 | 0 |
| 249            | 206 | 141 | 85  | 32 | 0 |

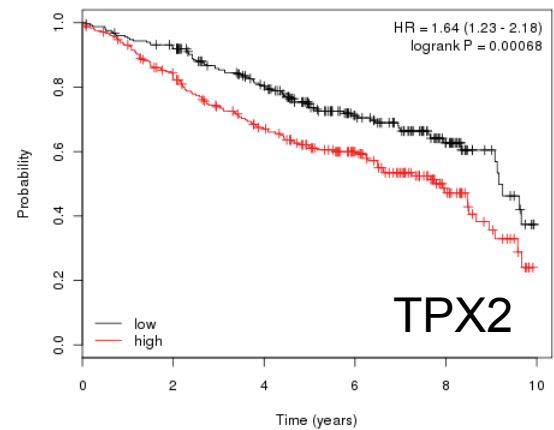

| number at risk |     |     |     |    |   |
|----------------|-----|-----|-----|----|---|
| 249            | 226 | 172 | 100 | 43 | 0 |
| 248            | 198 | 140 | 93  | 32 | 0 |

Supplement: Figure S4 — Individual prognostic performance of AURKA, FOXM1, TOP2A and TPX2 in breast cancer datasets as compared to CIN4 signature (groups split at median expression). (PDF) [file pone.0056707.s004.pdf]

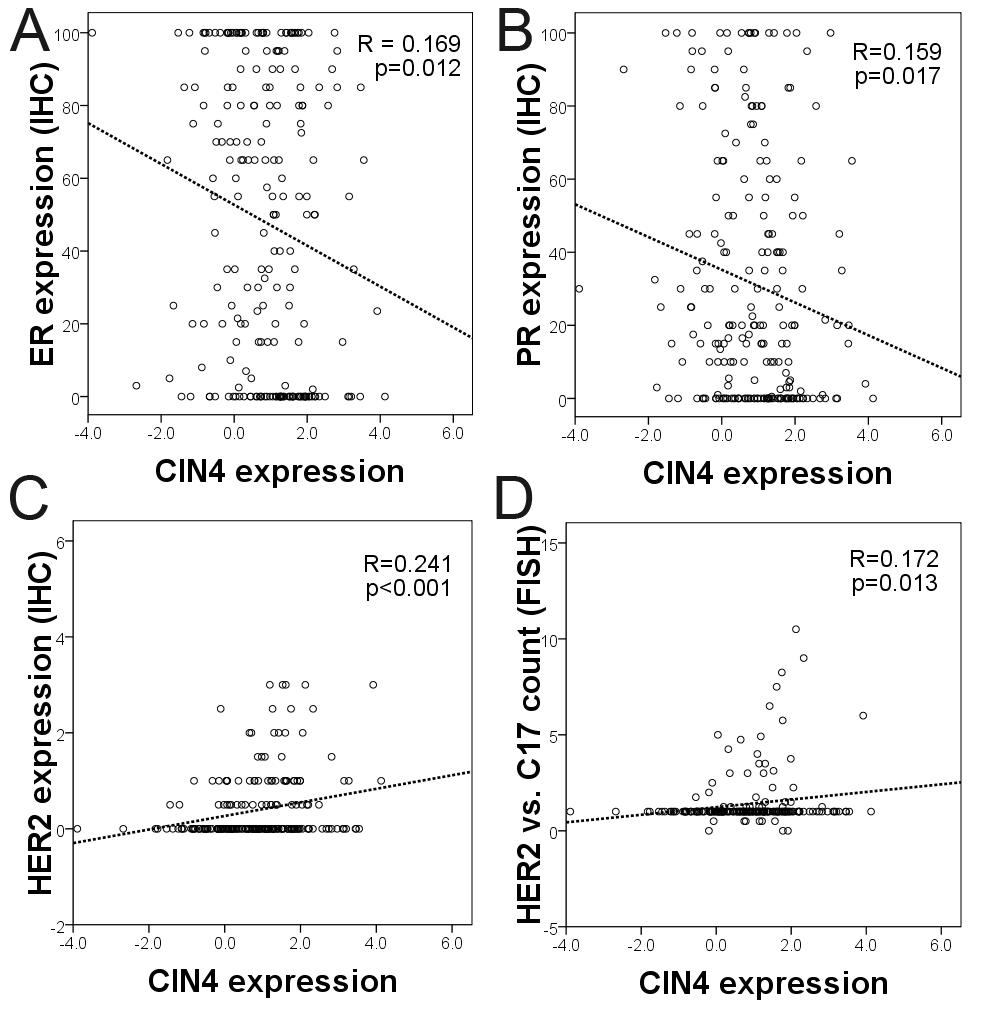

Supplement: Figure S5 — Correlation of CIN4 and markers used in the determination of immunophenotype. CIN4 and A) ER, B) PR, and C) HER2 expression, and D) HER2/chromosome 17 score determined by FISH. (TIF) [file pone.0056707.s005.tif]

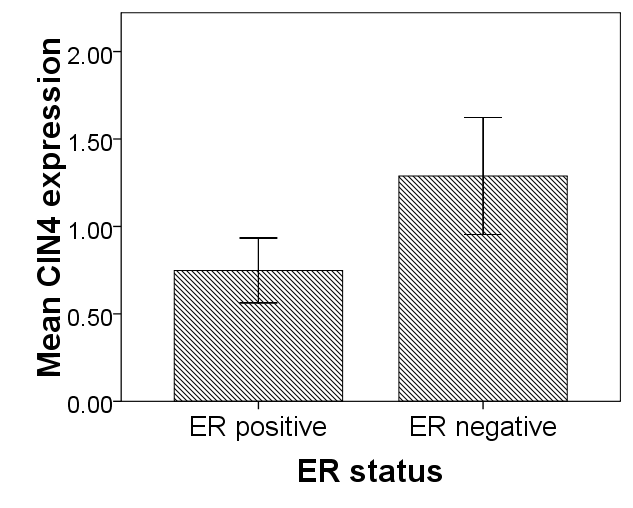

Supplement: Figure S6 — CIN4 expression in ER-negative and ER-positive tumors. (TIFF) [file pone.0056707.s006.tiff]
